# Antidepressant drug use in Europe: past consumption, prescribing patterns and forecast until 2030

**DOI:** 10.1007/s11096-025-02078-9

**Published:** 2026-03-10

**Authors:** Lilly Josephine Bindel, Roland Seifert

**Affiliations:** Institute of Pharmacology, Hannover Medical School, 30625 Hannover, Germany

**Keywords:** Antidepressants, Depression, Treatment, Europe, Consumption, Forecast

## Abstract

**Introduction:**

Mental disorders represent a significant global burden, with a high proportion of depressive disorders. Antidepressants (ADs) are the most prescribed drugs for treating mental disorders, with broad indications, e.g. depression, anxiety disorders, and off-label use.

**Aim:**

This study assesses current and historical changes in AD consumption in 25 European countries, predicts future developments, and further analyses ATC subgroup consumption in 13 countries.

**Method:**

Consumption data for ADs (ATC code N06A) were collected from the OECD Data Explorer. Subgroup data came from publicly available sources. The time span covered is 1980 to 2024. Changes in defined daily doses per 1000 population per day (DID) were analysed, and projections to 2030 were made using Auto Regressive Integrated Moving Average models. Treatment coverage for depressive disorder prevalence was also calculated.

**Results:**

In 2023, AD consumption ranged from 26.3 DID (Latvia) to 164.7 DID (Iceland). Use increased in nearly all countries, except Luxembourg (− 0.3 DID between 2013 and 2022). Increases ranged from + 5.9% (Austria) to + 157.8% (Latvia). Forecasts predict further increases in most countries (+ 0.1 to + 80.9%), with decreases projected for Hungary, Austria, the UK, and Luxembourg (− 3.4 to − 21.7%). Selective serotonin reuptake inhibitors (SSRIs) are the most used subgroup, with mixed developments. The second most used is miscellaneous, with growing shares in most countries. Non-selective monoamine reuptake inhibitors (NSMRIs) are declining, while monoamine oxidase inhibitors (MAO-Is) and monoamine oxidase A inhibitors (MAOA-Is) have very low shares. Northern Europe shows the highest AD use versus Eastern Europe the lowest. All regions primarily use SSRIs. Northern Europe has a higher miscellaneous share, while Eastern Europe uses more NSMRIs.

**Conclusion:**

AD use has increased and is projected to continue rising in most countries, with changing subgroup preferences. The dominance of SSRIs reflects first-line treatment recommendations. Increased miscellaneous use may indicate more individualised treatment and better tolerability. NSMRIs are declining due to adverse effects, and MAO-Is as well as MAOA-Is are rarely used due to safety concerns. Prescribing patterns vary across regions, influenced by health systems, cultural differences, stigma, and healthcare resources.

**Supplementary Information:**

The online version contains supplementary material available at 10.1007/s11096-025-02078-9.

## Impact statements

- A comprehensive overview of AD use in Europe is provided, revealing substantial differences in prescribing patterns and treatment coverage
- Regional disparities and drug class preferences are discussed to support rational prescribing
- The findings provide a basis for developing policies and improving treatment strategies for mental health conditions

## Introduction

Depression is one of the most common mental disorders [1, 2], with a prevalence of 4% worldwide and 4.3% in Central Europe [3, 4]. According to the Global Burden of Disease study, depressive disorders have the highest burden of all mental disorders, both globally and in Europe [5]. ADs are the most commonly used pharmacological treatments for mental disorders [2], with indications extending beyond depression, for example anxiety disorders, and are often used off-label [6–8]. Despite improvements in treatment, the burden of these disorders has increased in recent decades [9], as has their incidence [10].

Previous studies have documented divergent and potentially irrational prescribing practices across European countries and regions in various therapeutic areas, including the overuse of antibacterials, thyroid hormones, and antipsychotics, versus the underuse of lithium [11–14]. However, many existing studies focus on analysing past changes for individual countries [14–19], and insights on AD prescribing at the European level remain limited. A more comprehensive understanding of regional differences and influencing factors in antidepressant use is needed to support effective treatment and reduce the burden of disease.

### Aim

The aim of this study is to analyse past and current changes in the consumption of ADs (ATC code N06A) and their subgroups (Table S1) across European countries, and to provide an outlook on future developments up to 2030. This study provides a comprehensive European overview, examining both the overall volume and the proportional use of different AD classes. It also investigates regional variations and assesses potential factors influencing prescribing, and seeks to identify patterns of undertreatment and inappropriate drug choices in order to highlight opportunities for intervention and to promote rational prescribing. These insights aim to support the treatment of mental disorders, thereby reducing the burden of disease on both patients and healthcare systems.

## Method

### Setting and data

This study analyses the consumption of ATC N06A (ADs) and the subgroups ATC N06AA (NSMRIs), N06AB (SSRIs), N06AF (MAO-Is), N06AG (MAOA-Is), and N06AX (miscellaneous) [20]. The data covers 25 European countries, and is based on the OECD Data Explorer [21]. For 13 European countries, more detailed data about ATC subgroups were found in data provided by national authorities [22–47]. If only provisional data was provided for the latest year by the OECD, this assumption has been included. The data was stored in Excel without any data clean-up being required.

Data was reported in defined daily doses per 1000 inhabitants per day (DID), representing annual consumption, and spanning from 1980 to 2024. Where only DDD prescriptions were available, population data [48, 49] was used to calculate DID. For the regional and overall values, the median was calculated. The regions were separated partly based on geographical considerations, as well as the effort to include a comparable number of countries in each region. Its allocation is visualised in Figure S1. The maps were created with mapchart.net.

Table S2 outlines methodology differences, such as whether hospital sectors were included. While most data reflect the community sector, some countries include both. Exemplarily, in Denmark, the hospital sector has a consumption volume of 0.97 DID recently [26].

### Prediction of future consumption with ARIMA models

The autoregressive integrated moving average (ARIMA) model was chosen for its effectiveness in forecasting drug use developments [11, 50, 51] (further information in the supplemental Method). The Augmented Dickey-Fuller (ADF) test assessed stationarity [52], and model selection was based on the Bayesian Information Criterion (BIC). An individual ARIMA model was applied to each data set. Outliers were introduced for the years where the OECD data mentioned a ‘time break’ [21, 53], as well as in 2020 because of the COVID pandemic. Missing data has been treated as an omission.

Parameters were determined using Python in Google Colab, and forecasts were generated in SPSS [54]. Projections extend to 2030 with 95% confidence intervals. Forecast reliability was graded as ‘good’, ‘moderate’, or ‘poor’ based on metrics such as stationary R^2^, R^2^, MAPE, and MaxAPE [55]. A more detailed explanation for the assessment of fit metrics can be found in the supplement.

### Calculation of treatment coverage for ADs and depressive disorder

AD treatment coverage for depressive disorders was calculated to relate AD use to the country-specific prevalence of depressive disorders in Europe [56]. Prevalence data from 2021 was used, except for Luxembourg (2020). Coverage below 100% suggests underuse, while a coverage above 100% is more difficult to interpret and may indicate a tendency towards overtreatment.

### Ethics approval

This research was conducted according to the guidelines of good scientific practice of the Hannover Medical School (https://www.mhh.de/en/research/good-scientific-practice. The Hannover Medical School follows the guidelines of the German Research Foundation (Deutsche Forschungsgemeinschaft, DFG). No ethics approval is required because publicly available information is used as a data source.

## Results

### Consumption of ADs in the past and present

The latest published consumption data range from 26.3 DID (2023) in Latvia to 164.7 DID (2023) in Iceland (Table 1). In the last decade (since 2013), consumption increased in almost all countries for which sufficient data were available. Only in Luxembourg, the consumption decreased from 2013 until 2022 (− 0.6% relative; − 0.3 DID absolute).

**Table 1:** Past and current consumption of ADs

Country	DID in 2013	Last reported DID (year)	Relative change DID since 2013 (%)	Absolute change DID since 2013	Change last decade (since 2013)
Iceland	113.7	164.7 (2023)	44.9	51.0	↗
Portugal	87.5	154.4 (2023)	76.5	66.9	↗
United Kingdom	82.3	135.6 (2022)	64.8	53.3	↗
Sweden	84.3	117.8 (2023)	39.7	33.5	↗
Spain	65.2	101.5 (2023)	55.7	36.3	↗
Denmark	80.0	96.2 (2023)	20.3	16.2	↗
Belgium	72.1	88.2 (2022)	22.3	16.1	↗
Finland	69.4	85.2 (2021)	22.8	15.8	↗
Greece	44.7	76.5 (2023)	71.1	31.8	↗
Czech Republic	49.0	73.3 (2022)	49.6	24.3	↗
Germany	53.1	69.1 (2023)	30.1	16.0	↗
Slovenia	53.3	68.4 (2022)	28.3	15.1	↗
Norway	56.9	63.5 (2023)	11.6	6.6	↗
Austria	59.6	63.1 (2022)	5.9	3.5	↗
France	51.8	61.1 (2022)	18.0	9.3	↗
Luxembourg	53.4	53.1 (2022)	− 0.6	− 0.3	↘
Netherlands	43.3	50.8 (2023)	17.3	7.5	↗
Estonia	21.4	47.5 (2023)	122.0	26.1	↗
Italy	39.0	47.1 (2023)	20.8	8.1	↗
Slovakia	35.2	47.1 (2022)	33.8	11.9	↗
Lithuania	24.7	40.1 (2022)	62.3	15.4	↗
Croatia	26.7	39.4 (2023)	47.7	12.7	↗
Poland	–	39.1 (2023)	–	–	↗
Hungary	27.5	30.5 (2023)	10.9	3.0	↗
Latvia	10.2	26.3 (2023)	157.8	16.1	↗
Median	53.2	63.5	32.0	15.9	↗

The last reported consumption in DID and the relative changes for the last decade (2013–2023) are shown. Countries are listed in descending order of its last reported consumption

Regarding the regional AD consumption among European countries (Table 2), Northern Europe has the highest mean (median) (90.7 DID), followed by Southern (76.5 DID) and Western Europe (67.3 DID). Below the average (63.5 DID) are Central (68.4 DID) and Eastern Europe (47.1 DID).

**Table 2:**
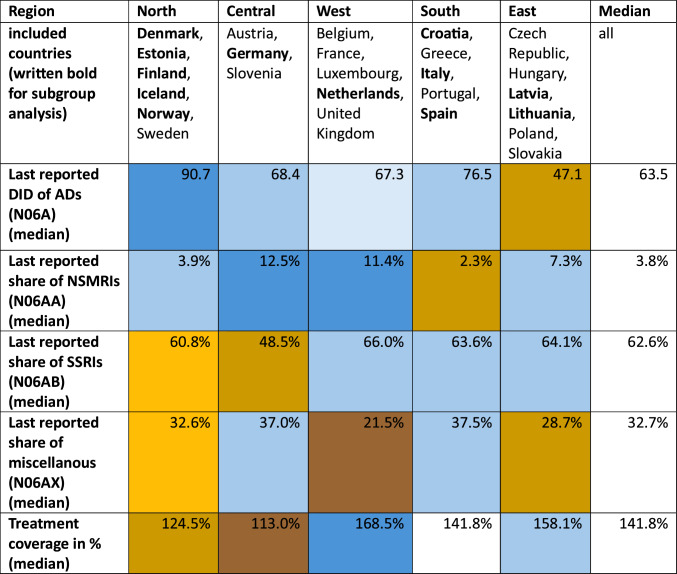
Assessment of study-defined European geographic regions

For each region, DID, shares of the three most frequently used ATC subgroups (N06AA/ NSMRIs, N06AB/ SSRIs, N06AX/ miscellaneous), and treatment coverages of depression prevalence were compared. Countries shown in bold had available ATC subgroup data and were therefore included in calculating the proportion of subgroup use. All countries within each region were included in calculating regional DID consumption. The median was used consistently for all regional calculations. Values above the regional average are shown in blue, and values below the regional average are shown in brown

### Consumption of AD subgroups (NSMRIs, SSRIs, MAO-Is, MAOA-Is, miscellaneous) in the past and present

In NSMRIs (ATC N06AA), e.g. amitriptyline, the last reported consumption ranges from 0.5 DID in Croatia to 8.7 DID in Germany (Table S3 and S5). Increasing developments are depicted for the three countries Norway, Spain and Latvia, and decreasing developments for the five countries Estonia, Lithuania, Germany, Croatia and Denmark (Table S6 and S2).

The last reported consumption of SSRIs (ATC N06AB), e.g. fluoxetine, ranges from 10.3 DID for Latvia to 122.6 DID for Iceland (Table S3 and S6). Seven from eight countries show increasing changes over the last ten years (+ 3.7 to + 14.4 DID) (Table S4 and S3). The exception is Norway, which recorded a slight decrease (− 0.6 DID absolute; − 1.7% relative).

The subgroups MAO-Is (ATC N06AF), e.g. phenelzine, and MAOA-Is (ATC N06AG), e.g. moclobemide, have a comparably low consumption and are not reported by some countries. Recent consumption of N06AF ranges from 0 to 0.5 DID (Table S3, S7 and S8), and for N06AG between 0 and 0.1 DID. Decreasing or stable changes are reported by most countries, with only Germany reporting an increase for both subgroups. However, given their marginal use, changes in consumption volume are more likely to be discreet fluctuations than meaningful developments.

For miscellaneous (ATC N06AX), e.g. mirtazapine, recent consumption ranges from 4 DID for Latvia to 41.3 DID for Iceland (Table S3 and S9). Eight countries show an increasing change.

### Distribution of subgroup proportions in AD use

In addition to consumption volumes, it is interesting to see how the subgroups of ADs are distributed and what shifts have occurred over the considered period. Detailed results can be found in Tables 3, S5–S9, and in Figure S2.

**Table 3:** Last reported distributional use in % for ATC subgroups of ADs

Country (year)	NSMRIs (N06AA) (%)	SSRIs (N06AB) (%)	MAO-Is (N06AG) (%)	MAOA-Is (N06AF) (%)	Miscellaneous (N06AX) (%)
Iceland (2023)	2.7	73.1	0.1	–	24.1
Italy (2023)	2.3	68.8	–	–	29.1
Netherlands (2023)	11.4	66.0	0.1	1.0	21.5
Lithuania (2022)	3.0	64.2	–	–	32.8
Denmark (2024)	3.6	64.0	0.0	0.1	32.2
Latvia (2018)	11.6	63.9	–	–	24.5
Croatia (2023)	1.3	61.2	0.0	0.0	37.5
Norway (2023)	6.5	60.8	0.1	0.0	32.6
Estonia (2023)	3.9	59.4	0.0	0.0	36.7
Spain (2023)	3.4	57.9	0.0	–	38.8
Finland (2020)	4.8	54.7	0.4	–	40.1
Germany (2023)	12.5	48.5	0.2	0.2	37.0

Countries are sorted in descending order of the largest share of N06AB. Belgium is not listed because the last available data point is from 2014

SSRIs are the group with the largest share of consumption in all 8 countries with recent data, ranging from 48.5 to 73.1% (Table S6). Over the last decade, decreasing changes up to − 11.8% can be observed in six of eight countries, including Estonia, Latvia, Lithuania, Norway, Spain and Croatia. In contrast, increases up to 4.4% are depicted for the two countries Denmark and Germany. Western Europe has the highest share (66.0%), followed by East (64.1%), South (63.6%), North (60.8%), and Central (48.5%) Europe (Table 2).

The second most used group is miscellaneous, with a share of 21.5–40.1% (Table S9). In all 8 countries, the share increased, ranging from + 0.9 to + 14.6%. The highest proportion is depicted for South Europe (37.5%), followed by Central (37.0%), North (32.6%), East (28.7%) and West (21.5%) Europe.

The third most used ATC group are NSMRIs, with shares ranging from 1.3 to 12.5% in recent years (Table S6). Eight countries reported a decreasing share, with percentage points ranging from − 0.5 to − 8.0% over the last decade. Central Europe has the largest share with 12.5%, followed by West (11.4%), East (7.3%), North (3.9%) and South (2.3%) Europe.

MAO-Is and MAOA-Is have a very low share, ranging from 0 to 0.4% (Table S7, S8). Developments were decreasing or stable in almost all countries. The only country with an increase is Germany with + 0.02 DID increase in MAOA-Is within the last decade.

### Forecast of AD consumption until 2030

To assess future developments in AD use, DID (ATC N06A) were predicted to 2030 using ARIMA models. Further information can be found in Tables S10–S13. An evaluation of the model reliability is available in the supplement.

**Fig. 1:**
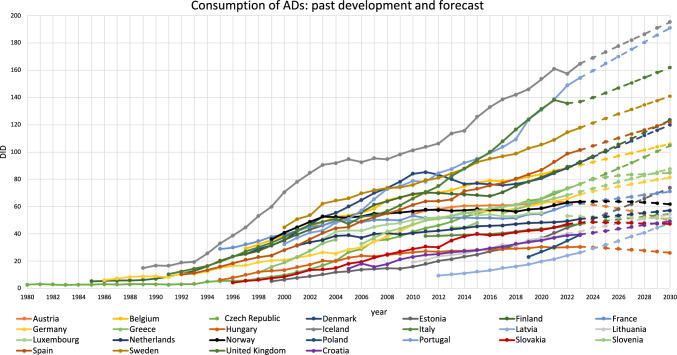
Past development and prediction of DID for ADs (ATC N06A). Forecasts are highlighted as dotted lines

In 2030, AD consumption is predicted to range from 26.0 DID in Hungary to 195.2 DID in Iceland (Fig. 1, Table S10). Projections were possible for 24 of 25 countries for which data were provided, a model for Poland could not be built due to insufficient data.

The predicted DID are increasing in most countries (+ 0.1 to 80.9%), while decreases are predicted for four countries: Hungary (− 14.6% relative change), Austria (− 21.7%), Luxembourg (− 3.4%), and Norway (− 2.8%) (Table S10). This means that the increases observed in recent years will continue in 20 countries, while for the other three declining countries, the prediction suggests a changing development. Luxembourg, which is predicted to decline, already experienced a slight decline in the last decade.

### Treatment coverage of depressive disorder by ADs

In 2021, treatment coverage varies from 43.9% in Latvia to 473.8% in Iceland (Table 4). Countries with coverage below 100%, indicating potential undertreatment, include Latvia, Lithuania, Hungary, Estonia and Italy. All other countries have coverages exceeding 100%.

**Table 4:** Treatment coverage of depressive disorder prevalence by ADs

Country	Compared year	DID of ADs [85]	Prevalence of depressive disorder [56] (%)	Treatment coverage of depression prevalence by ADs (%)
Latvia	2021	21.5	4.9	*43.9*
Lithuania	2021	37.4	5.4	*69.3*
Hungary	2021	29.8	3.4	*87.6*
Estonia	2021	40.7	4.6	*88.5*
Italy	2021	44.6	4.5	***99.1***
Czech Republic	2021	37.1	3.5	***106.0***
Netherlands	2021	48.5	4.4	**110.2**
Greece	2021	70.6	6.3	**112.1**
Poland	2021	30.5	2.7	**113.0**
France	2021	57.6	4.8	**120.0**
Slovakia	2021	44.7	3.4	**131.5**
Norway	2021	61.1	4.4	**138.9**
Luxembourg	2020	55.3	3.9	**141.8**
Germany	2021	64.0	4.2	**152.4**
Finland	2021	85.2	5.2	**163.8**
Spain	2021	92.7	5.5	**168.5**
Austria	2021	63.5	3.6	**176.4**
Slovenia	2021	66.1	3.7	**178.6**
Croatia	2021	69.4	3.5	**198.3**
Belgium	2021	86.2	4.3	**200.5**
Denmark	2021	84.6	4.2	**201.4**
Sweden	2021	108.9	5.1	**213.5**
Portugal	2021	138.8	5.8	**239.3**
United Kingdom	2021	138.2	5.2	**265.8**
Iceland	2021	161.1	3.4	**473.8**

The comparison year, the DID and prevalence in 2020 or 2021 and the treatment coverage in % are given. Coverage is highlighted in italics for below 100%, bold italics for around 100% and blod for above 100%. Countries are sorted in ascending order of the treatment coverage

Western Europe has a treatment coverage of 168.5% (median), followed by East (158.1%), South (141.8%), North (124.5%), and Central (113.5%) Europe (Table 2).

## Discussion

### Patterns of consumption and distribution in ADs

ADs belong to the most frequently used drug classes for mental disorders [57]. Since many years, a strong increase in AD use has been reported for most European countries [8, 58–60], which is also reflected in observed and predicted changes in DID (Table 1, Fig. 1). Several aspects contribute to this, including improved diagnostic and healthcare capacity [61], increasing incidence and awareness of mental disorders [62–66], development and expansion of clinical guidelines [67, 68], destigmatisation of mental disorders [69], patent expirations resulting in decreasing DDD costs [70], and pharmaceutical industry promotion [65]. There has also been a broadening of the indications from major depression to other conditions such as anxiety, obsessive–compulsive disorder and post-traumatic stress disorder [8, 17], more frequent off-label use [7, 62] and an increase in pharmacological treatment of young and old patients [71].

While the overall use is predicted to increase, some countries are expected to decline (Table S10). In cases such as Austria and Hungary, where significant reductions are forecast despite previous upward developments, it is debatable whether these results reflect an implausible ARIMA model rather than a substantiated development. A similar situation may apply to Norway, where consumption has increased slowly but steadily in recent years; however, the ARIMA model appears rather unreliable (see Table S12). In contrast, Luxembourg is the only country showing an actual decline in past data, which is predicted to continue. However, this may also be a methodological artefact, possibly influenced by the circumstance that the most recent data point refers to 2022 and may have been an effect of the COVID pandemic [72], with no clear evidence explaining Luxembourg’s' exceptional downward conversion.

There has been a shift in the use of ATC subgroups within ADs (Tables 4, 6, 10 and S2-S6). While SSRIs dominated in the early 2010s’ and still have the largest share (Table 3), recent data suggest an increasing use of miscellaneous [64, 73] (Table S3–S4). In contrast, the shares of NSMRIs have been decreasing, while changes in consumption vary (Table S3, S5). NSMRIs are considered similar effective than SSRIs, but are associated with adverse drug effects, especially for elderly patients [6, 74]. The use of MAO-Is and MAOA-Is remain low and stable (Table S7–S8), reflecting their role in niche indications such as treatment-resistant depression [75–77] because of safety concerns such as serious adverse drug effects and dietary restrictions [78, 79]. SSRIs remain a first-line treatment for major depression, valued for their balance of efficacy and safety [6, 80, 81]. However, adverse drug effects or lack of efficacy lead some patients to discontinue or switch treatment [81]. The miscellaneous group includes newer drugs and has increased in use (Table S4) due to broader indications, better tolerability in certain patients and expanding guideline recommendations [6, 62, 82]. These patterns are observed in the majority of countries analysed [73], suggesting a general pattern.

### National specifics: explanation of deviating characteristics in Europe

Iceland is the country with the highest consumption of ADs and treatment coverage of depression (164.7 DID; 473.8%) (Tables 1 and 4). Several factors contribute to this, such as the cultural acceptance of ADs [83], limited access to and lower cost than other treatments such as psychotherapy [84]. Furthermore, Iceland has the highest share of SSRIs (73.1%) and the lowest share for miscellaneous (24.1%) (Table 3). This may be explained by lifted restrictions on the dispensing of SSRIs in 2009 [85] and them being preferred due to their efficacy and fewer adverse drug effects [86].

In contrast, Latvia has the lowest consumption and treatment coverage of ADs (26.3 DID; 43.9%), being attributed to underdiagnosis of depression and anxiety disorders [87], negative public attitudes and stigmatization of mental disorders, limited access to health services and financial burden for patients [88–90].

The strong growth of DID in Portugal (+ 66.9%) (Table 1) can be explained by improvements in diagnosis and access to treatment, prolonged use and a special reimbursement scheme with reduced co-payments for patients [71, 91, 92]. In contrast, Luxembourg experienced a slight decrease (− 0.6%), possibly due to a shift towards non-pharmacological treatment [93].

Germany has the highest proportion of NSMRIs (12.5%), the highest proportion of miscellaneous (37.0%) and the lowest proportion of SSRIs (48.5%) (Table 3). Influencing factors may be the warning of adverse drug effects for SSRIs by national authorities and their controversial debate in Germany [6], which may have led to a preference for other drugs. This discrepancy between the low national share vs. the recommendation of SSRIs as a first-line treatment in clinical guidelines [94] points towards an influence of cultural factors on prescribing behaviour [2, 95, 96].

### Geographical patterns of prescribing behaviour

**Fig. 2:**
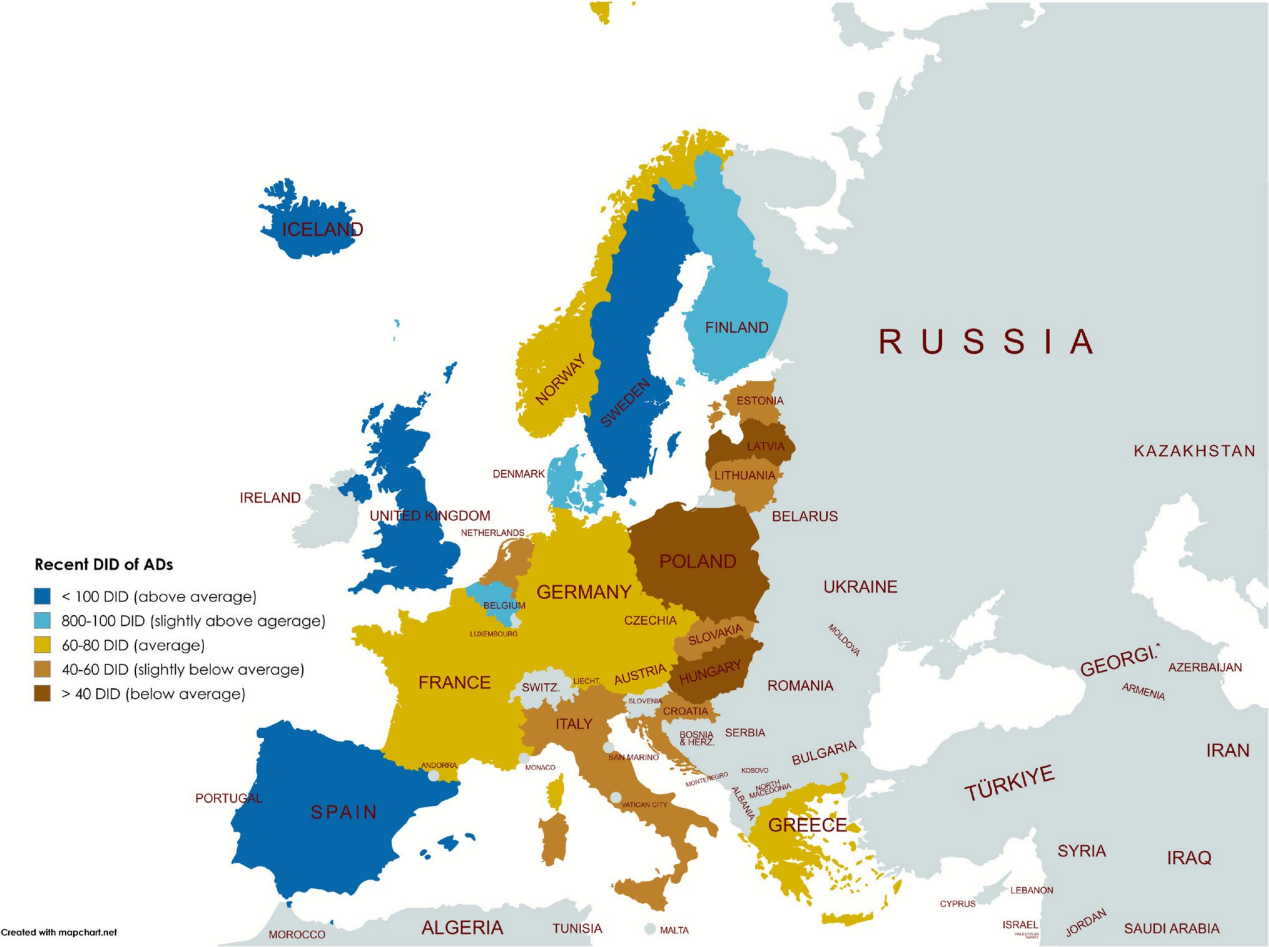
Geographical distribution of AD consumption in the analysed countries. The most recently reported prescriptions in DID are projected. Consumption ranges are highlighted in colour: dark brown for strongly below average, light brown for slightly below average, yellow for average, light blue for slightly above average and dark blue for strongly above average. Countries for which no data were available are shown in grey

Prescribing patterns vary regionally and within countries (Tables 1, 2, 3, 4 and Fig. 2). Recent DID of ADs, changes in recent years, projected changes and treatment coverages for depressive disorder differ substantially. These differences reflect disparities in health care infrastructure, driven more by general health expenditure than by the cost of ADs per defined daily dose [58, 61, 71]. Other contributing factors include the presence of national mental health programmes, the financial burden on patients, prescribing practices and cultural differences such as stigma of mental disorders or acceptance of pharmacological treatment [88–90], as well as methodological restrictions (see section ‘Limitations’). Access to non-pharmacological treatments such as psychotherapy also varies, often limited in rural areas like those in Iceland [84].

In addition, the use of ATC groups is also unevenly distributed (Figure S3). SSRIs are the dominant AD subgroup in all regions and are consistently recommended as first-line treatment for depression [6, 97, 98]. However, Central Europe (represented by Germany) has a comparatively low proportion of SSRI use, due to higher use of newer alternatives (miscellaneous) [6]. NSMRIs have minimal use in Northern and Southern Europe, while Western and Eastern Europe show a continued reliance on NSMRIs with a lower use of miscellaneous (Figure S3).

## Limitations

The study is based on data from the OECD and national health authorities, with varying observation periods and methodologies, as well as the inclusion of provisional values of the OECD Data Explorer [21]. Furthermore, a different allocation of countries in geographical regions is possible. This results in restrictions in the granularity of comparisons for consumption volumes, and restrictions in the ARIMA forecasts.

Key assumptions in this study are that a patient's dosage corresponds to a defined daily dose (DDD) prescription, that every depressive disorder has to be treated with ADs, and that ADs are only used for depressive disorder. However, these assumptions may be simplified, resulting in uncertainties in presented consumption data, treatment coverage, and assessment. Individual treatment dosages of DDD vary in clinical practice, with a tendence of the need of higher treatment dosages than the assigned DDDs, e.g. in sertraline with the DDD corresponding to the starting dose but a clinical use up to four times the DDD [20, 99], resulting in a potential overestimation of treatment coverage. Nevertheless, DID are considered a valuable tool in the assessment of changes and international comparisons [97]. Despite depression being the condition where the largest share of ADs is prescribed for [100], a considerable amount of ADs is used for other indications, e.g. being a first-choice treatment in anxiety disorders, as well as their off-label use [7, 8, 17, 62, 101, 102] (Table 5), resulting in a potential overestimation of treatment coverage. The lack of differentiation of consumption volumes by indication is a recognized but yet persistent problem [103]. Therefore, the interpretation of the treatment coverages comes with uncertainties and is to consider in comparison with other countries rather than an exact value.

**Table 5:** Comparison of the analysed depressive disorders versus the most common mental disorders, anxiety disorders [122]

Comparison of depressive versus anxiety disorders	Depressive disorders	Anxiety disorders
Prevalence: Central Europe, Eastern Europe, and Central Asia, all ages, both sexes, 2023 (upper; lower) [123]	4.30% (5.17%; 3.65%)	4.62% (5.96%; 3.56%)
Pharmacological treatment options [101, 124, 124–126]	ADs (several drug classes)	ADs (several drug classes), benzodiazepines (second-choice, short-term treatment)

While these conditions have a comparable prevalence and ADs are considered as the first-choice pharmacological treatment in both [101]. Anxiety disorders are frequently treated by other drug classes like benzodiazepines [127]. Because depressive disorder is the indication where the largest share of consumption is used for [100], depressive disorders were analysed in the context of ADs

## Conclusion

The use of ADs in European countries follows a general development. Overall, consumption has increased [58, 59, 71] and is predicted to continue to rise in most countries (Fig. 1 and Table 1). Among the ATC groups, NSMRIs are becoming increasingly obsolete, as reflected by a decreasing share (Table S4). On the other hand, miscellaneous has become more popular due to advantages in tolerability and expanded guideline recommendations [6, 73, 82], as evidenced by increasing DID and shares (Table S9). Nevertheless, SSRIs remain the most commonly used drug group [104], with the highest proportion in all countries [72] (Table 3). This is consistent with the recommendation of SSRIs as first-line treatment for depression [6, 80, 81, 105].

Differences in AD consumption among European regions can partly be explained by influencing factors such as the access to mental health services, national programs, the availability of psychotherapy, and cultural stigma [58, 61, 71, 88, 106, 107]. Prescribing differences extend beyond ADs to other drug classes, such as antipsychotics, antibacterials and thyroid hormones, particularly between Northern and Eastern Europe [11–14], suggesting long-standing patterns.

A rational treatment of depression is essential, consisting of a multimodal approach with pharmacological treatment as a component for moderate to severe depression, with an optimal drug selected based on the individual patient profile, safety aspects, efficacy and cost [108–110]. Appropriate treatment is very important as depression is the most common mental disorder [2] and a major burden [111] both for the individual patient and at the population level, with increasing incidence [62, 112].

Assessing the appropriateness of AD use is challenging due to several factors. They are not only prescribed for depression but also for other mental health conditions and off-label uses [17, 71, 113]. Additionally, combination therapy involving multiple ADs complicates evaluation [114, 115]. Mild forms of depression may not require pharmacological treatment [98], raising concerns about potential overuse [6, 113, 116]. However, literature reports underuse in depressive persons for all the examined countries and regions [117–119], while the findings of our study suggest definitive underuse in Latvia, Lithuania, Hungary and Estonia (treatment coverage below 100%) (Table 4). Importantly, treatment coverage exceeding 100% cannot be interpreted as evidence of overuse, due to the broad application of ADs for multiple indications and the lack of information on specific indications in drug consumption statistics. The challenges of multi-indication use and the inability to differentiate consumption by indication have also been noted in other prescribing analyses [95].

It has become increasingly clear that ADs and other psychiatric medications are prescribed for a wide range of conditions. Not only have ADs shown efficacy in depressive disorders, they have also demonstrated therapeutic potential in other mental health conditions, just as various psychotropic drugs have in depression. These overlaps highlight shortcomings in the diagnosis and treatment of mental health conditions. Consequently, a new approach is necessary: one that focuses on symptoms rather than diagnoses, also known as 'reverse pharmacology'. This includes reforming the categorisation of the 'International Statistical Classification of Diseases and Related Health Problems' (ICD) and ATC classifications, as well as moving towards a mechanistically oriented nomenclature [120, 121].

To support the development of more rational and effective treatment strategies, several proposals for action were outlined from the authors for national health authorities and corresponding stakeholders:

1. **Strengthen diagnostic and treatment infrastructure** by improving accessibility, adequate resource allocation, and increasing the availability of qualified health professionals.
2. **Reduce stigma associated with mental disorders** through awareness campaigns and public education.
3. **Provide guidance** for professionals and patients by up-to-date guidelines and information material.
4. **Establish guideline-adherence** by restrictive measures and training programs.
5. **Support new approaches for treatment and drug development**, including the use of “**reverse pharmacology**”, where drugs may serve as diagnostic tools.
6. **Revise existing disease classifications** like ICD and ATC to prioritise symptom-based categorization, and establish the use of a **new mechanistically orientated nomenclature**.
7. **Monitor developments,** collect comprehensive data on prescribing and treatment, and invest in research.

Further research is needed to confirm and generalise our findings. The analyses should be extended to other countries and geographical regions, and a more specific insight into country-specific regional differences should be obtained. In particular, an analysis of the consumption of ATC level 4 would provide further insights. A projection of the development of DID and shares for ATC subgroups is also valuable for a more differentiated insight. Additionally, the appropriateness of treatment with ADs needs to be assessed in detail. The analysis of prescribing behaviour in an international perspective should be extended to other classes of medicines.

## Supplementary Information

Below is the link to the electronic supplementary material.

Supplementary file1 (DOCX 2736 kb)

## Data Availability

All source data for this study are available upon reasonable request from the authors.

## References

[1] Liu Q, He H, Yang J, Fengf X, et al. Changes in the global burden of depression from 1990 to 2017: findings from the Global Burden of Disease study. J Psychiatric Res. 2020;126:134–40. 10.1016/j.jpsychires.2019.08.002. 10.1016/j.jpsychires.2019.08.002 31439359

[2] Pan American Health Organization (PAHO). World Mental Health Day: Depression, the Most Common Mental Disorder. News. PAHO 2012. 2012. https://www.paho.org/en/news/9-10-2012-world-mental-health-day-depression-most-common-mental-disorder. Accessed 9 Apr 2025.

[3] Our World in Data. Mental illness prevalence, World, 2021. Data source: IHME, Global Burden of Disease. 2024. https://ourworldindata.org/grapher/mental-illnesses-prevalence. Accessed 13 Apr 2025.

[4] Łaszewska A, Österle A, Wancata J, et al. Prevalence of mental diseases in Austria : systematic review of the published evidence. Wien Klin Wochenschr. 2018;130(3–4):141–50. 10.1007/s00508-018-1316-1. 29368240 10.1007/s00508-018-1316-1 PMC5816100

[5] Our World in Data. Burden of disease from each category of mental illness, World, 2021. Data source: IHME, Global Burden of Disease. 2024. https://ourworldindata.org/grapher/burden-disease-from-each-mental-illness. Accessed 13 Apr 2025.

[6] Ludwig W, Mühlbauer M, Seifert R. Arzneiverordnungs-report 2023. 1st ed. Berlin, Heidelberg: Springer; 2024.

[7] Sheffler ZM, Patel P, Abdijadid S. Antidepressants. Statpearls Publishing 2023, Treasure Island (FL). https://www.ncbi.nlm.nih.gov/books/NBK538182/30844209

[8] Martella M, Minutiello E, Gianino MM. Patterns of antidepressant and anxiolytic use and spending in 14 European countries (2012–2021): a comprehensive time series analysis. Health Serv Insights. 2024;17:11786329241282526. 10.1177/11786329241282526. 39386264 10.1177/11786329241282526 PMC11462615

[9] Liu J, Liu Y, Ma W, et al. Temporal and spatial trend analysis of all-cause depression burden based on Global Burden of Disease (GBD) 2019 study. Sci Rep. 2024;14:12346. 10.1038/s41598-024-62381-9. 38811645 10.1038/s41598-024-62381-9 PMC11137143

[10] Santomauro DF, Herrera AM, Shadid J, et al. (COVID-19 Mental Disorders Collaborators). Global prevalence and burden of depressive and anxiety disorders in 204 countries and territories in 2020 due to the COVID-19 pandemic. The Lancet. 2021;398(10312):1700–12. 10.1016/S0140-6736(21)02143-7. 10.1016/S0140-6736(21)02143-7 PMC8500697 34634250

[11] Bindel LJ, Seifert R. Most European countries will miss EU targets on antibacterial use by 2030: historical analysis of European and OECD countries, comparison of community and hospital sectors and forecast to 2040. Naunyn-Schmiedebergs Arch Pharmacol. 2025. 10.1007/s00210-025-03887-5. 39960558 10.1007/s00210-025-03887-5 PMC12350445

[12] Bindel LJ, Seifert R. Aware classification analysis for European countries with ARIMA forecasts to assess prescribing patterns and ‘One Health’ targets. Naunyn-Schmiedebergs Arch Pharmacol. 2025. 10.1007/s00210-025-04121-y. 40220024 10.1007/s00210-025-04121-y PMC12511250

[13] Bindel LJ, Seifert R. Long-term forecasting and evaluation of medicine consumption for the ATC class H with a focus on thyroid hormones in OECD countries using ARIMA models. Naunyn-Schmiedebergs Arch Pharmacol. 2025. 10.1007/s00210-025-03930-5. 40029386 10.1007/s00210-025-03930-5 PMC12350577

[14] Bindel LJ, Seifert R. Evidence of lithium underuse in bipolar disorder: analysis of lithium and antipsychotic consumption, prediction of future trends, regional disparities and indicators of rational and inappropriate use in Europe. Naunyn-Schmiedebergs Arch Pharmacol. 2025. 10.1007/s00210-025-04389-0. 40580313 10.1007/s00210-025-04389-0 PMC12678625

[15] Oscoz-Irurozqui M, Villani L, Martinelli S, et al. Trend analysis of antidepressant consumption in Italy from 2008 to 2022 in a public health perspective. Sci Rep. 2025;15(1):12124. 10.1038/s41598-025-96037-z. 40204785 10.1038/s41598-025-96037-z PMC11982533

[16] Cebron Lipovec N, Anderlic A, Locatelli I. General antidepressants prescribing trends 2009–2018 in Slovenia: a cross-sectional retrospective database study. Int J Psychiatry Clin Pract. 2022;26(4):401–5. 10.1080/13651501.2022.2057331. 35416749 10.1080/13651501.2022.2057331

[17] Arias LH, Lobato CT, Ortega S, et al. Trends in the consumption of antidepressants in Castilla y León (Spain). Association between suicide rates and antidepressant drug consumption. Pharmacoepidemiol Drug Saf. 2010;19(9):895–900. 10.1002/pds.1944. 20712020 10.1002/pds.1944

[18] Madeira L, Queiroz G, Henriques R. Prepandemic psychotropic drug status in Portugal: a nationwide pharmacoepidemiological profile. Sci Rep. 2023;13:6912. 10.1038/s41598-023-33765-0. 37106018 10.1038/s41598-023-33765-0 PMC10139661

[19] Heald AH, Stedman M, Davies M, et al. Antidepressant prescribing in England: patterns and costs. Primary Care Compan CNS Disorders. 2020;22(2):19m02552. 10.4088/PCC.19m02552. 10.4088/PCC.19m02552 32302071

[20] World Health Organization (WHO). ATC/DDD Index. N Nervous system. WHO. 2024. Last accessed: March 31 2025. https://atcddd.fhi.no/atc_ddd_index/?code=N06A&showdescription=no. Accessed 31 Mar 2025.

[21] Organisation for Economic Co-operation and development (OECD). OECD Data Explorer. Pharmaceutical consumption. OECD. 2025. https://data-viewer.oecd.org/?chartId=2c0ba8fa-1b08-4694-babf-43ae35b8659e. Accessed 4 Apr 2025.

[22] Vermeylen M, Hans G, Baeyens JP et al. (Comité d’évaluation des pratiques médicales en matière de médicaments). Tableaux de bord pharmaceutices. Délivrances pharmaceutiques dans le secteur ambulant 2014. Institut national d'assurance maladie-invalité (INAMI). 2015. https://www.inami.fgov.be/SiteCollectionDocuments/pharma_tableau_de_bord_rapport_2014.pdf. Accessed 25 Mar 2025.

[23] Draganić P, Škribulja M, Oštarčević S et al. Potrošnja lijekova u Hrvatskoj 2018–2022. Agency for Medicinal Products and Medicals Devices (HALMED). 2023. https://www.halmed.hr/fdsak3jnFsk1Kfa/publikacije/Potrosnja-lijekova-u-Hrvatskoj-2018-2022.pdf. Accessed 25 Mar 2025.

[24] Draganić P, Škribulja M, Oštarčević S et al. Potrošnja lijekova u Hrvatskoj 2013–2017. Agency for Medicinal Products and Medicals Devices (HALMED). 2018. https://www.halmed.hr/fdsak3jnFsk1Kfa/publikacije/Potrosnja-lijekova-u-Hrvatskoj-2013-2017.pdf. Accessed 25 Mar 2025.

[25] Draganić P, Žeželić S, Šarinić VM et al. Potrošnja lijekova u Hrvatskoj 2007–2012. Agency for Medicinal Products and Medicals Devices (HALMED). 2014. https://www.halmed.hr/fdsak3jnFsk1Kfa/publikacije/Potrosnja_lijekova_u_Hrvatskoj_2007-2012.pdf. Accessed 25 Mar 2025.

[26] Sundhedsdatastyrelsen. Medstat.dk. The Danish Health Data Authority. 2024. Copenhagen. https://www.medstat.dk/en. Accessed 25 Mar 2025.

[27] ANDMEBAAS. Statistics on medicines. ATC-N: Nervous system. Health Statistics and Health Research Database. 2025. https://statistika.tai.ee/pxweb/en/Andmebaas/Andmebaas__06Ravimistatistika__01Ravimistatistika/ATC-N.px/. Accessed 25 Mar 2025.

[28] Savaikis L, Seilis A, Gailite E et al. Baltic statistics on medicines 2016–2018. State Medicines Control Agency of Lithuania. 2019. Vilnius, Lithuania. ISBN 978-609-462-139-0. https://www.zva.gov.lv/sites/default/files/2020-01/Baltic%20statistics_3rd%20edition.pdf. Accessed 17 Feb 2025.

[29] Seilis A, Gailite E, Rootslane L et al. Baltic Statistics on Medicines 2013–2015. 2nd edition. Latvian State Agency of Medicines 2016. Riga, Latvia. ISBN 978-9934-8602-2-5. https://www.zva.gov.lv/sites/default/files/2018-05/Baltic%20Statistics%20on%20Medicines%202013%20-%202015.pdf. Accessed 17 Feb 2025.

[30] Rootslane L, Laius O, Sepp J et al. Baltic Statistics on Medicines 2010–2012. Estonian State Agency of Medicines 2013. Tartu, Estonia. ISBN 978-9949-33-397-4. https://www.zva.gov.lv/sites/default/files/2018-05/BS_2013.pdf. Accessed 18 Feb 2025.

[31] Finnish Medicines Agency (FIMEA). Drug consumption statistics. Drug consumption in years 2018–2021. FIMEA. 2021. http://raportit.nam.fi/raportit/kulutus/laakekulutus_e.html. Accessed 19 Feb 2025.

[32] Wissenschaftliches Institut der AOK (WIdO). PharMaAnalyst. WidO. 2024. https://arzneimittel.wido.de/PharMaAnalyst/?1. Accessed 25 Mar 2025.

[33] Directorate of Health. Lyfjanotkun á Íslandi. Eftir ATC flokkunarkerfi lyfja. Directorate of Health 2025. 2025. https://app.powerbi.com/view?r=eyJrIjoiZmRiMGJkNmMtZWQ4NC00NmUzLTlkY2UtZTQ0NDk5ZjZmMDE2IiwidCI6Ijc2NGEzMDZkLTBhNjgtNDVhZC05ZjA3LTZmMTgwNDQ0N2NkNCIsImMiOjh9. Accessed 22 Mar 2025.

[34] Italian Medicines Agency. National Report on Medicines Use in Italy. Year 2023. The Medicines Utilisation Monitoring Centre. 2024. Rome. ISBN: 979-12-80335-37-1. https://www.aifa.gov.it/documents/20142/2594020/AIFA_Rapporto_OsMed_2023_EN.pdf. Accessed 24 Mar 2025.

[35] Russo P, Cangini A, Fortinguerra F et al. National Report on Medicines Use in Italy. Year 2022. The Medicines Utilisation Monitoring Centre. Rome: Italian Medicines Agency; 2023. ISBN: 979‐12‐80335‐31‐9. https://www.aifa.gov.it/documents/20142/2143103/Rapporto-OsMed-2022_EN.pdf. Accessed 24 Mar 2025.

[36] Cangini A, Fortinguerra F, Pierantozzi A et al. National Report on Medicines Use in Italy. Year 2021. The Medicines Utilisation Monitoring Centre 2022. Rome. Italian Medicines Agency. ISBN: 979‐12‐80335‐26‐5. https://www.aifa.gov.it/documents/20142/1740782/Rapporto-OsMed-2021_EN.pdf. Accessed 24 Mar 2025.

[37] Cangini A, Fortinguerra F, Pierantozzi A et al. National Report on Medicines Use in Italy. Year 2020. The Medicines Utilisation Monitoring Centre 2021. Rome: Italina Medicines Agency; 2021. ISBN: 979-12-80335-17-3. https://www.aifa.gov.it/documents/20142/1542390/Rapporto-OsMed-2020_EN.pdf. Accessed 24 Mar 2025.

[38] Trotta F, Traversa G, Altamura G et al. National Report on Medicines Use in Italy. The Medicines Utilisation Monitoring Centre 2020. Rome: Italian Medicines Agency; 2019. ISBN: 979­12­80335­00­5. https://www.aifa.gov.it/documents/20142/241052/OsMed_2019_Eng.pdf. Accessed 24 Mar 2025.

[39] Trotta F, Filippo AD, Vito AD et al. National Report on Medicines Use in Italy. The Medicines Utilisation Monitoring Centre 2020. Rome: Italian Medicines Agency; 2018. https://www.aifa.gov.it/documents/20142/241052/OsMed_2018_Eng.pdf. Accessed 22 Mar 2025.

[40] Trotta F, Filippo AD, Vito AD et al. National Report on Medicines Use in Italy. The Medicines Utilisation Monitoring Centre 2019. Rome: Italian Medicines Agency; 2017. https://www.aifa.gov.it/documents/20142/241052/OsMed_2017_eng.pdf. Accessed 22 Mar 2025.

[41] Gailite E, Seilis A, Zake A. Statistics on medicines consumption 2012. State Agency of Medicines. 2013. https://www.zva.gov.lv/sites/default/files/2018-05/Zalu_paterina_statistika_2012-20130604.pdf. Accessed 22 Mar 2025.

[42] State Medicines Control Agency of Lithuania. Medicines consumption 2022. State Medicines Control Agency of Lithuania. 2023. https://vvkt.lrv.lt/media/viesa/saugykla/2023/9/5gjLnBtWc7s.docx. Accessed 23 Mar 2025.

[43] Dutch Healthcare Institute. GIPdatabank.nl. Dutch Healthcare Institute. 2024. https://www.gipdatabank.nl/databank?infotype=g&label=00-totaal&tabel=B_01-basis&geg=ddd&item=N. Accessed 23 Mar 2025.

[44] Folkehelseinstituttet. Norwegian Prescription Database. The Norwegian Institute of Public Health. 2021. https://www.norpd.no/. Accessed 23 Mar 2025.

[45] Olsen K, Skoufa II, Bakken GV et al. Drug Consumption in Norway 2019–2023. Data from Norwegian Drug Wholesales Statistics. Folkehelseinstituttet. The Norwegian Institute of Public Health 2024. Oslo, Norway. ISBN: 978-82-8406-459-8. https://www.fhi.no/contentassets/b0802ad9303347b682cf6a8fa701ec91/legemiddelforbruket-i-norge-2019-2023-rapport-2024.pdf. Accessed 23 Mar 2025.

[46] Ministerio de Sanidad. Consumo de Productos Farmacéuticos. Datos de consumo de recetas médicas del SNS según clasificación Anatómica-Terapéutica-Química (ATC). Ministerio de Sanidad. 2025. https://www.sanidad.gob.es/areas/farmacia/consumoMedicamentos/ATC/home.htm. Accessed 23 Mar 2025.

[47] Socialstyrelsen. Statistikdatabas för läkemedel. Socialstyrelsen. 2025. https://sdb.socialstyrelsen.se/if_lak/val.aspx. Accessed 23 Mar 2025.

[48] STATBEL. Population by place of residence, nationality (Belgian/non-Belgian), marital status, age and gender. STATBEL. 2024. Last accessed: March 26 2025. https://bestat.statbel.fgov.be/bestat/crosstable.xhtml?view=1b9e219b-0387-4a70-880a-dc5eccaa244c. Accessed 26 Mar 2025.

[49] Eurostat. Database. Population (national level). European Union. Eurostat. 2025. https://ec.europa.eu/eurostat/databrowser/view/tps00001/default/table?lang=en&category=t_reg.t_reg_dem. Accessed 26 Mar 2025.

[50] Hyndman RJ, Athanasopoulos G. Forecasting: principles and practice. 2nd edition. OTexts 2021. Melbourne, Australia. OTexts.com/fpp2. Accessed 24 Sept 2024.

[51] Nau, R. Linear regression models. Statistical forecasting: notes on regression and time series analysis. Fuqua School of Business, Duke University; 2020. https://people.duke.edu/~rnau/rsquared.htm. Accessed 28 May 2025.

[52] Dickey DA, Fuller WA. Distribution of the estimators for autoregressive time series with a unit root. J Am Stat Assoc. 1979;74(366):427–31. 10.2307/2286348.

[53] Organisation for Economic Co-operation and development (OECD). OECD Health Statistics 2024. Definitions, Sources and Methods. Total pharmaceutical consumption by DDDs. OECD; 2024. https://stats.oecd.org/wbos/fileview2.aspx?IDFile=6f2bfdca-6a41-4b50-9b47-b0a1a7d12a1e. Accessed 4 Apr 2025.

[54] IBM. Time Series Model. IBM SPSS Modeler. IBM; 2021. https://www.ibm.com/docs/hr/spss-modeler/saas?topic=SS3RA7_sub/modeler_mainhelp_client_ddita/clementine/timeseries_modelnode.htm. Accessed 29 May 2025.

[55] Bindel LJ, Seifert R. Long-term forecast for antibacterial drug consumption in Germany using ARIMA models. Naunyn Schmiedebergs Arch Pharmacol. 2025. 10.1007/s00210-024-03721-4. 39754681 10.1007/s00210-024-03721-4 PMC12125074

[56] Our World in Data. Depressive disorders prevalence, 2021. Data source: IHME, Global Burden of Disease; 2024. https://ourworldindata.org/grapher/depressive-disorders-prevalence-ihme?region=Europe. Accessed 13 Apr 2025.

[57] Lunghi C, Dugas M, Leclerc J, et al. Global prevalence of antidepressant drug utilization in the community: protocol for a systematic review. BMJ Open. 2022;12(5):e062197. 10.1136/bmjopen-2022-062197. 35641008 10.1136/bmjopen-2022-062197 PMC9157341

[58] Lewer D, O’Reilly C, Mojtabai R, et al. Antidepressant use in 27 European countries: associations with sociodemographic, cultural and economic factors. Br J Psychiatry. 2018;207(3):221–6. 10.1192/bjp.bp.114.156786. 10.1192/bjp.bp.114.156786 26159603

[59] Mikulic M. Percentage change in consumption of antidepressant drugs between 2010 and 2020 in Europe, by country. Statista. Pharmaceutical Products & Market. Survey by OECD, published by Euronews. Statista. 2025. https://www.statista.com/statistics/1446772/consumption-change-of-antidepressants-in-the-last-decade-in-europe-by-country/#:~:text=Across%20the%20analyzed%20European%20countries,108%20and%20101%20percent%2C%20respectively. Accessed 31 Mar 2025.

[60] Peano A, Calabrese F, Pechlivanidis K, et al. International trends in antidepressant consumption: a 10-year comparative analysis (2010–2020). Psychiatr Q. 2025. 10.1177/11786329241282526. 40029558 10.1007/s11126-025-10122-0 PMC12213856

[61] Chen S, Ford TJ, Jones PB, et al. Prevalence, progress, and subgroup disparities in pharmacological antidepressant treatment of those who screen positive for depressive symptoms: a repetitive cross-sectional study in 19 European countries. Lancet Regional Health Eur. 2022;17:100368. 10.1016/j.lanepe.2022.100368. 10.1016/j.lanepe.2022.100368 PMC8969158 35373171

[62] Pazzagli L, Reutfors J, Lucian E, et al. Increased antidepressant use during the COVID-19 pandemic: findings from the Friuli Venezia Giulia Region, Italy, 2015–2020. Psychiatry Res. 2022;315:114704. 10.1016/j.psychres.2022.114704. 35830755 10.1016/j.psychres.2022.114704 PMC9245333

[63] World Health Organization (WHO). COVID-19 pandemic triggers 25% increase in prevalence of anxiety and depression worldwide. News. WHO; 2022. https://www.who.int/news/item/02-03-2022-covid-19-pandemic-triggers-25-increase-in-prevalence-of-anxiety-and-depression-worldwide. Accessed 4 Apr 2025. PMC9998057 35414629

[64] Yu Z, Zhang J, Zheng Y, et al. Trends in antidepressant use and expenditure in six major cities in China from 2013 to 2018. Front Psychiatry. 2020. 10.3389/fpsyt.2020.00551. 32765307 10.3389/fpsyt.2020.00551 PMC7378967

[65] Moreno-Agostino D, Wu Y, Daskalopoulou C, et al. Global trends in the prevalence and incidence of depression: a systematic review and meta-analysis. J Affect Disord. 2021;281:235–43. 10.1016/j.jad.2020.12.035. 33338841 10.1016/j.jad.2020.12.035

[66] Foulkes L, Andrews JL. Are mental health awareness efforts contributing to the rise in reported mental health problems? A call to test the prevalence inflation hypothesis. New Ideas Psychol. 2023;69:101010. 10.1016/j.newideapsych.2023.101010.

[67] Boysen G, Doonan A. Expansion of the concept of mental disorder in the DSM-5. J Mind Behav. 2014; 35:225–244. https://www.researchgate.net/publication/289721837_Expansion_of_the_concept_of_mental_disorder_in_the_DSM-5. Accessed 29 Oct 2025.

[68] Zhou W, Yu Y, Yang M, et al. Policy development and challenges of global mental health: a systematic review of published studies of national-level mental health policies. BMC Psychiatry. 2018;18:138. 10.1186/s12888-018-1711-1. 29776356 10.1186/s12888-018-1711-1 PMC5960139

[69] Haslam N, Tse JS. Public awareness of mental illness: mental health literacy or concept creep? Australas Psychiatry. 2025;33(1):18–20. 10.1177/10398562241292202. 39402888 10.1177/10398562241292202 PMC11804130

[70] Huskamp HA, Donohue JM, Koss C, et al. Generic entry, reformulations and promotion of SSRIs in the US. Pharmacoeconomics. 2008;26(7):603–16. 10.2165/00019053-200826070-00007. 18563951 10.2165/00019053-200826070-00007 PMC2719790

[71] Barbato A, Vallarina M, Rapisarda F et al. Access to mental health care in Europe. EU compass for action on mental health and well-being. 2016. https://health.ec.europa.eu/system/files/2016-12/ev_20161006_co04_en_0.pdf. Accessed 5 Apr 2025.

[72] Organisation for Economic Co-operation and Devleoppment (OECD). Evaluation of Luxembourg's COVID-19 Response: Learning from the Crisis to Increase Resilience. Paris: OECD Publishing; 2022. 10.1787/2c78c89f-en

[73] Viola R, Benko R, Nagy G, et al. National trend of antidepressant consumption and its impact on suicide rate in Hungary. Pharmacoepidemiol Drug Saf. 2008;17(4):401–5. 10.1002/pds.1574. 18314926 10.1002/pds.1574

[74] Santarsieri D, Schwartz TL. Antidepressant efficacy and side-effect burden: a quick guide for clinicians. Drugs Context. 2015;4:212290. 10.7573/dic.212290. 26576188 10.7573/dic.212290 PMC4630974

[75] Thase ME. MAOIs and depression treatment guidelines. J Clin Psychiatry. 2012;73(7):e24. 10.4088/JCP.11096tx4c. 22901357 10.4088/JCP.11096tx4c

[76] Thomas SJ, Shin M, McInnis MG, et al. Combination therapy with monoamine oxidase inhibitors and other antidepressants or stimulants: strategies for the management of treatment-resistant depression. Pharmacotherapy. 2015;35(4):433–49. 10.1002/phar.1576. 25884531 10.1002/phar.1576

[77] Birkenhager TK, Heijnen WT. Monoamine oxidase inhibitors: seriously underused in the treatment of major depression. Acta Psychiatr Scand. 2024;150(6):497–9. 10.1111/acps.13753. 39227148 10.1111/acps.13753

[78] Sub Laban T, Saadabadi A. Monoamine Oxidase Inhibitors (MAOI). Treasure Island (FL): StatPearls Publishing; 2023. Last accessed: October 28 2025. https://www.ncbi.nlm.nih.gov/books/NBK539848/30969670

[79] Hirsch M, Birnbaum RJ, Roy-Byrne PP et al. Monoamine oxidase inhibitors (MAOIs): Pharmacology, administration, safety, and side effects. UpToDate. 2025. https://www.uptodate.com/contents/monoamine-oxidase-inhibitors-maois-pharmacology-administration-safety-and-side-effects#topicContent. Accessed 4 Apr2025.

[80] Chu A, Wadhwa R. Selective Serotonin Reuptake Inhibitors. Treasure Island (FL): StatPearls Publishing; 2023. Last accessed: October 28 2025. https://www.ncbi.nlm.nih.gov/books/NBK554406/32119293

[81] National Health Service (NHS). Overview-Selective serotonin reuptake inhibitors (SSRIs). NHS; 2021. https://www.nhs.uk/mental-health/talking-therapies-medicine-treatments/medicines-and-psychiatry/ssri-antidepressants/overview/. Accessed 4 Apr 2025.

[82] Schwasinger-Schmidt TE, Macaluso M. Other Antidepressants. Handb Exp Pharmacol. 2019;250:325–55. 10.1007/164_2018_167. 30194544 10.1007/164_2018_167

[83] Sigurdsson E, Olafsdóttir T, Gottfredsson M. Public views on antidepressant treatment: lessons from a national survey. Nord J Psychiatry. 2008;62(5):374–8. 10.1080/08039480801984156. 18752102 10.1080/08039480801984156

[84] Vilhelmsson A. Depression and antidepressants: a Nordic perspective. Front Public Health Sec Epidemiol. 2013. 10.3389/fpubh.2013.00030. 10.3389/fpubh.2013.00030 PMC3854846 24350199

[85] Thengilsdottir G, Gardarsdottis H, Almarsdottis AB, et al. The association between lifting an administrative restriction on antidepressant dispensing and treatment patterns in Iceland. Health Policy. 2013;111(2):193–9. 10.1016/j.healthpol.2013.03.002. 23548199 10.1016/j.healthpol.2013.03.002

[86] Laursen M, Jensen A, Schwartson R et al. (NOMENSCO group on Medicine Statistics). Antidepressants prevalence. Nordic Health & Welfare Statistics; 2024. https://nhwstat.org/health/pharmaceutical-products/prevalence/antidepressants-prevalence. Accessed 5 Apr 2025.

[87] Harro J, Aadamsoo K, Rootslane L, et al. Comparison of psychotropic medication use in the Baltic countries. Nord J Psychiatry. 2020;74(4):301–6. 10.1080/08039488.2019.1707283. 31889460 10.1080/08039488.2019.1707283

[88] Styraite G, Buka AA, Sarskute A et al. Depression Management in Primary Care in Lithuania and Latvia. Biol Psychiatry Psychopharmacol 2020; 22(1). https://biological-psychiatry.eu/wpcontent/uploads/2020/06/BPP_May2020_17to23.pdf. Accessed 31 Oct 2025.

[89] Depression Scorecard. Spotlight on Latvia. Depressioncare.eu 2022. 2022. https://www.depressioncare.eu/latvia.html?utm. Accessed 5 Apr 2025.

[90] China-CEE Institute. Latvia social briefing: Mental Health in Latvia: Unveiling Challenges and Charting the Path Forward. Institute of Economics at the Latvian Academy of Sciences 2023. Vol. 65, No. 3 (LVA). https://china-cee.eu/2023/10/27/latvia-social-briefing-mental-health-in-latvia-unveiling-challenges-and-charting-the-path-forward/?utm. Accessed 31 Oct 2025.

[91] Estrela M, Herdeiro MT, Ferreira PL, et al. The use of antidepressants, anxiolytics, sedatives and hypnotics in Europe: focusing on mental health care in Portugal and prescribing in older patients. Int J Environ Res Public Health. 2020;17(22):8612. 10.3390/ijerph17228612. 33228203 10.3390/ijerph17228612 PMC7699589

[92] TPN/Lusa. Increased antidepressant use “not all negative”. The Portugal News. 2022. https://www.theportugalnews.com/news/2022-10-09/increased-antidepressant-use-not-all-negative/70997?utm. Accessed 5 Apr 2025.

[93] Malmendier-Muehlschlegel A, Power NC. Mental health services in Luxembourg: an overview. BJPsych Int. 2022;19(3):72–4. 10.1192/bji.2021.58. 36287821 10.1192/bji.2021.58 PMC9540651

[94] Fugger G, Bartova L, Fabbri C, et al. The sociodemographic and clinical profile of patients with major depressive disorder receiving SSRIs as first-line antidepressant treatment in European countries. Eur Arch Psychiatry Clin Neurosci. 2022;272:715–27. 10.1007/s00406-021-01368-3. 34989830 10.1007/s00406-021-01368-3 PMC9095529

[95] Bindel LJ, Seifert R. Trends, prescribing patterns and projections of antiseizure drug use in Europe. Epilepsy Res. 2025;218:107689. 10.1016/j.eplepsyres.2025.107689. 41151402 10.1016/j.eplepsyres.2025.107689

[96] Seifert R. Basic knowledge of pharmacology. 1st edition. Switzerland AG: Springer; 2019. 10.1007/978-3-030-18899-3

[97] World Health Organization (WHO). Defined Daily Dose (DDD). Definition and general considerations. ATC-DDD Toolkit. WHO 2025. https://www.who.int/tools/atc-ddd-toolkit/about-ddd. Accessed 25 May 2025.

[98] Naber D, Bullinger M. Should antidepressants be used in minor depression? Dialogues Clin Neurosci. 2018;20(3):223–8. 10.31887/DCNS.2018.20.3/dnaber. 30581292 10.31887/DCNS.2018.20.3/dnaber PMC6296391

[99] Singh HK, Saadabadi A. Sertraline. StatPearls Publishing, Treasure Island (FL); 2023. Last updated: February 13 2023. https://www.ncbi.nlm.nih.gov/books/NBK547689/. Accessed 28 Oct 2025.

[100] Camacho-Arteaga LF, Gardasdottir H, Ibannez L, et al. Indications related to antidepressant prescribing in the Nivel-PCD database and the SIDIAP database. J Affect Disord. 2022;303:131–7. 10.1016/j.jad.2022.02.001. 35134393 10.1016/j.jad.2022.02.001

[101] Butlen-Ducuing F, Haberkamp M, Aislaitner G, et al. The new European Medicines Agency guideline on antidepressants: a guide for researchers and drug developers. Eur Psychiatry. 2023;67(1):e2. 10.1192/j.eurpsy.2023.2479. 38098366 10.1192/j.eurpsy.2023.2479 PMC10790230

[102] Bandelow B, Werner AM, Kopp I, et al. The German guidelines for the treatment of anxiety disorders: first revision. Eur Arch Psychiatry Clin Neurosci. 2022;272(4):571–82. 10.1007/s00406-021-01324-1. 34609587 10.1007/s00406-021-01324-1 PMC8490968

[103] Wertheimer AI, Santella TM. Problems using the Defined Daily Dose (DDD) as a statistical basis for drug pricing and reimbursement. International Federation of Pharmaceutical Manufacturers & Associations (IFPMA); 2007. Last updated: 2007. https://www.ifpma.org/wp-content/uploads/2023/01/i2023_IFPMA_DDD_2007_EN.pdf. Accessed 28 Oct 2025.

[104] MedlinePlus. Commonly prescribed antidepressants and how they work. National Institute of Mental Health; 2023. https://magazine.medlineplus.gov/article/commonly-prescribed-antidepressants-and-how-they-work#:~:text=Different%20types%20of%20antidepressants%20affect,to%20treat%20insomnia%20and%20anxiety. Accessed 9 Apr 2025.

[105] Kendrick T, Taylor D, Johnson CF. Which first-line antidepressant? Br J Gen Pract. 2019;69(680):114–5. 10.3399/bjgp19X701405. 30819733 10.3399/bjgp19X701405 PMC6400617

[106] Dlouhy M. Mental health policy in Eastern Europe: a comparative analysis of seven mental health systems. BMC Health Serv Res. 2014;14:42. 10.1186/1472-6963-14-42. 24467832 10.1186/1472-6963-14-42 PMC3908346

[107] Mitchell G, Murphy E, Bojarcz S et al. Is an EU-wide approach to the mental health crisis necessary? FEPS Policy Study. 2023. ISBN: 9782931233061. https://feps-europe.eu/wp-content/uploads/2023/03/PS-Is-an-EU-wide-approach-to-the-mental-health-crisis-necessary.pdf. Accessed 5 Apr 2025.

[108] Prescott D, White ND. When is pharmacotherapy initiation beneficial in patients with depressive disorders? Am J Lifestyle Med. 2017;11(3):220–2. 10.1177/1559827616686051. 30202334 10.1177/1559827616686051 PMC6125088

[109] Dunlop BW. Evidence-based applications of combination psychotherapy and pharmacotherapy for depression. Focus (American Psychiatric Publishing). 2016;14(2):156–73. 10.1176/appi.focus.20150042. 31975799 10.1176/appi.focus.20150042 PMC6519650

[110] Karyotake E, Smit Y, Henningsen KH, et al. Combining pharmacotherapy and psychotherapy or monotherapy for major depression? A meta-analysis on the long-term effects. J Affect Disord. 2016;194:144–52. 10.1016/j.jad.2016.01.036. 26826534 10.1016/j.jad.2016.01.036

[111] Proudman D, Greenberg P, Nellesen D. The growing burden of major depressive disorders (MDD): implications for researchers and policy makers. Pharmacoeconomics. 2021;39(6):619–25. 10.1007/s40273-021-01040-7. 34013439 10.1007/s40273-021-01040-7 PMC8134814

[112] Zhang Y, Jia X, Yang Y, et al. Change in the global burden of depression from 1990–2019 and its prediction for 2030. J Psychiatr Res. 2024;178:16–22. 10.1016/j.jpsychires.2024.07.05. 39106579 10.1016/j.jpsychires.2024.07.054

[113] Anmella G, Sanabra M, Primé-Tous M et al. Antidepressants overuse in primary care: Prescription trends between 2010 and 2019 in Catalonia. Revista de Psiquiatría y Salud Mental 2022. 10.1016/j.rpsm.2022.12.001 10.1016/j.rpsm.2022.12.001 37758595

[114] Palaniyappan L, Insole L, Ferrier N. Combining antidepressants: a review of evidence. Adv Psychiatr Treat. 2009;15(2):90–9. 10.1192/apt.bp.107.004820.

[115] Rush AJ, Roy-Byrne PP, Solomon D. Unipolar depression in adults: treatment with antidepressant combinations. UpToDate 2025. 2025. https://www.uptodate.com/contents/unipolar-depression-in-adults-treatment-with-antidepressant-combinations#H78562923. Accessed 13 Apr 2025.

[116] Jureidini J, Tonkin A. Overuse of antidepressant drugs for the treatment of depression. CNS Drugs. 2006;20(8):623–32. 10.2165/00023210-200620080-00002. 16863268 10.2165/00023210-200620080-00002

[117] Boehlen FH, Freigofas J, Herzog W, et al. Evidence for underuse and overuse of antidepressants in older adults: results of a large population-based study. Int J Geriatr Psychiatry. 2019;34(4):539–47. 10.1002/gps.5047. 30623499 10.1002/gps.5047

[118] Francia L, De Giorgi R, Lara E, et al. Treatment coverage for depression in the general Spanish population and the impact of the Covid-19 pandemic. Heliyon. 2024;10(11):e32594. 10.1016/j.heliyon.2024.e32594. 38961986 10.1016/j.heliyon.2024.e32594 PMC11219982

[119] Santomauro DF, Vos T, Whiteford HA, et al. Service coverage for major depressive disorder: estimated rates of minimally adequate treatment for 204 countries and territories in 2021. Lancet Psychiatry. 2024;11(12):1012–21. 10.1016/S2215-0366(24)00317-1. 39572105 10.1016/S2215-0366(24)00317-1 PMC11579305

[120] Seifert R, Schirmer B, Seifert J. How pharmacology can aid in the diagnosis of mental disorders. Naunyn Schmiedebergs Arch Pharmacol. 2025;398:1099–110. 10.1007/s00210-024-03413-z. 39230588 10.1007/s00210-024-03413-z PMC11825625

[121] Seifert R, Schirmer B. A simple mechanistic terminology of psychoactive drugs: a proposal. Naunyn-Schmiedebergs Arch Pharmacol. 2020;393:1331–9. 10.1007/s00210-020-01918-x. 32535698 10.1007/s00210-020-01918-x PMC7351828

[122] Javaid SF, Hashim IJ, Hashim MJ, et al. Epidemiology of anxiety disorders: global burden and sociodemographic associations. Middle East Curr Psychiatry. 2023;30:44. 10.1186/s43045-023-00315-3.

[123] Institute for Health Metrics and Evaluation (IHME). GBD Results. GBD 2023. University of Washington; 2025. Last updated: 2025. https://vizhub.healthdata.org/gbd-results/. Accessed 25 Oct 2025.

[124] Barnhill JW. Overview of anxiety disorders. Anxiety and stressor-related disorders. MSD Manual, Professional Version; 2024. Last updated: January 2024. https://www.msdmanuals.com/professional/psychiatric-disorders/anxiety-and-stressor-related-disorders/overview-of-anxiety-disorders. Accessed 28 Oct 2025.

[125] Centre for Addiction and Mental Health (CAMH). Depression: psychopharmacology. treating conditions and disorders. CAMH, Canada; 2019. Last updated: 2019. https://www.camh.ca/en/professionals/treating-conditions-and-disorders/depression/depression---treatment/depression---psychopharmacology. Accessed 28 Oct 2025.

[126] Brandt J, Bressi J, Lê ML, et al. Prescribing and deprescribing guidance for benzodiazepine and benzodiazepine receptor agonist use in adults with depression, anxiety, and insomnia: an international scoping review. eClinicalMedicine. 2024. 10.1016/j.eclinm.2024.102507. 38516102 10.1016/j.eclinm.2024.102507 PMC10955669

[127] Tanguay Bernard MM, Luc M, Carrier JD, et al. Patterns of benzodiazepines use in primary care adults with anxiety disorders. Heliyon. 2018;4(7):e00688. 10.1016/j.heliyon.2018.e00688. 29998202 10.1016/j.heliyon.2018.e00688 PMC6039319

