# Supplementary material for: Antidepressant drug use in Europe: past consumption, prescribing patterns and forecast until 2030
Source: Int J Clin Pharm. 2026 Mar 10;48(3):819–33. doi: 10.1007/s11096-025-02078-9 (PMC13176021; doi:10.1007/s11096-025-02078-9)

**Antidepressant drug use in Europe: Past consumption, prescribing patterns and forecast until 2030**

**Supplement**

**Lilly Josephine Bindel and Roland Seifert**

Supplemental Methods

**Forecast of consumption with ARIMA models**

The autoregressive integrated moving average (ARIMA) model was chosen for its ability to capture different components of a time series, including autoregressive (AR) behaviour, differentiation (I) and moving average (MA) components. The model is defined by its three parameters ARIMA(*p, d, q*). The autoregressive term (*p*) represents the number of past observations that influence the current forecast, with higher values increasing the reliance on historical data but potentially adding complexity. The differencing order (*d*) ensures stationarity by eliminating trends, although excessive differencing can lead to overfitting and loss of meaningful structure. The moving average term (*q*) determines how past errors affect the current forecast. This model is particularly well suited to data sets where past values significantly influence future developments [10-13, 49, 107].

**Assessment of fit metrics for the ARIMA forecasts**

The assessment of reliability is based on the following model fit metrics: stationary R-squared (which assesses the stationary part of the model), R-squared (the coefficient of determination), mean absolute percentage error (MAPE, which assesses how strongly the model varies from its original values), and maximum absolute percentage error (MaxAPE, which is similar to MAPE but expressed as a percentage) [13, 106]. A good fit is defined as stationary R-squared above 0.65, R-squared above 0.85, MAPE below 6 and MaxAPE below 15. Moderate fit includes R-squared between 0.4 and 0.65, R-squared between 0.6 and 0.84, MAPE between 7 and 20 and MaxAPE between 16 and 40. Poor fit is indicated by R-squared less than 0.4, R-squared less than 0.6, MAPE greater than 20 and MaxAPE greater than 40 [12,107]. The ARIMA model fit for each country is graded as “good”, “moderate” or “poor” based on the above-mentioned metrics.

The accuracy of the prediction is assessed using the upper and lower confidence limits (LCL and UCL). These values are related to a 'relative range' to evaluate the width of the confidence interval in relation to the predicted value. This is calculated for each country for the year 2030 as follows:

$$relative range in \%= \frac{UCL-LCL}{prediction} \times100$$

Given the relatively limited data set, the predictive power of the model is limited. Therefore, more emphasis should be placed on the reliability of trends rather than the precise prediction of specific values in the long term [10, 11]. Furthermore, the forecast is based on past data and therefore predicts future changes on the assumption that past trends will continue in the future. Changing circumstances or sudden events can't be foreseen.

Supplemental Results

**Forecast reliability**

In order to interpret the predicted antidepressant use, it is necessary to assess the performance of the ARIMA models. In the following, the reliability of the model is assessed by its fit metrics and the relative range of the 95% confidence interval in 2030, and classified as good, moderate or poor.

The fit metrics of each ARIMA model can be used to assess the ability of the forecast model to fit its curve to past data points (Table S8 and S9). In general, about the half of the models are deficient in terms of stationary R-squared, but perform comparatively well in terms of R-squared, MAPE and MaxAPE. Good fit metrics are considered for the 4 countries Italy, Latvia, Lithuania and Luxembourg. Moderate fit metrics are considered for all other countries. However, Poland could not be assessed due to the inability to develop a model.

The uncertainty of the forecast can be assessed by the relative range of the 95% confidence interval (presented by UCL and LCL in 2030), depicted in Table S9. If the range is narrow, the forecast is fairly certain, whereas a wide range means that the future development is uncertain. Narrow ranges below 20% are shown by 7 countries: Belgium, Iceland, Latvia, Lithuania, Portugal, Slovenia and Spain. Moderate ranges between 30% and 50% are found in the 11 countries Czech Republic, Estonia, France, Germany, Hungary, Italy, Luxembourg, the Netherlands, Sweden, the United Kingdom and Croatia. A wider range of over 50% is found in the 6 countries Austria, Denmark, Finland, Greece, Norway and Slovakia.

The assessment of the accuracy of the forecast model combines the fit metrics and the width of the confidence interval (Table S9). A well-fitted model has been assessed for Italy, Latvia, Lithuania and Luxembourg, represented by good fit metrics and a narrow confidence interval. A forecast that is reliable for trends but not as well-fitted as it could be considered reliable for exact values, assessed by good to moderate fit metrics and confidence interval widths below 50%, is considered for Belgium, Czech Republic, Estonia, France, Germany, Hungary, Iceland, the Netherlands, Portugal, Slovenia, Spain, Sweden, the United Kingdom and Croatia. An uncertain forecast, judged by a wide confidence interval width above 50%, is considered for Austria, Denmark, Finland, Greece, Norway and Slovakia.

In general, model reliability and forecast plausibility are not necessarily related. While the ARIMA model may fit past data well, this does not imply that past performance will continue into the future. Conversely, a model with comparatively poor fit metrics may provide a close and plausible forecast. Predicted consumption must be assessed critically, as ARIMA models provide a possible scenario by assuming that past developments will continue in the future, while they can't take into account changing circumstances [90].

Supplemental Tables

***Table S1:*** *ATC classification of antidepressants (ADs) and its subgroups. Given is the ATC Code, the covered drug group and examples.*

| **ATC code** | **Drug group** | **Examples [19]** |
| --- | --- | --- |
| **N06A** | Antidepressants (ADs) |  |
| **N06AA** | Non-selective monoamine reuptake inhibitors (NSMRIs) | amitriptyline, nortriptyline, imipramine, doxepin, clomipramine |
| **N06AB** | Selective serotonin reuptake inhibitors (SSRIs) | fluxetine, citalopram, sertraline, etoperidone, escitalopram |
| **N06AF** | Monoamine oxidase inhibitors, non-selective (MAO-Is) | nialamide, phenelzine, tranylcypromine, iproniazide |
| **N06AG** | Monoamine oxidase A inhibitors (MAOA-Is) | moclobemide |
| **N06AX** | Miscellaneous | venlafaxine, mirtazapine, trazodone, vortioxetine, agomelatine |

***Table S2****: Data availability for the analysed European countries for AD by the OECD Data Explorer (https://stats.oecd.org/wbos/fileview2.aspx?IDFile=6f2bfdca-6a41-4b50-9b47-b0a1a7d12a1e) and available national reports for ATC subgroups. Mentioned is the available time period and the covered health care sector. Further information about data methodology is provided for the national databases, while for the OECD Data Explorer, a comprehensive overview is available (https://stats.oecd.org/wbos/fileview2.aspx?IDFile=6f2bfdca-6a41-4b50-9b47-b0a1a7d12a1e).*

| **Country** | **Available years**  **(AD; ATC subgroups)** | **Covered health care sector** | **Weblink for ATC subgroups**  **(last accessed: April 13 2025)** | **Information about national data source methodology** |
| --- | --- | --- | --- | --- |
| **Austria** | 2010-2022;  not found | primary care | not found | - |
| **Belgium** | 1997-2022;  2006-2014 | primary care | https://www.inami.fgov.be/fr/statistiques/statistiques-des-medicaments/tableaux-de-bord-pharmaceutiques | includes reimbursed medications to non-hospitalized patients (exclusion of over-the-counter (OTC) prescriptions, non-reimbursed drugs, hospitalized patients) |
| **Czech Republic** | 1980-2022;  not found | primary and hospital care | not found | - |
| **Denmark** | 1997-2022;  1996-2024 | primary and hospital care | https://www.medstat.dk/en | includes all sales of medicines (outpatient and hospitalized sector; OTC drugs; reimbursed and non-reimbursed drugs) |
| **Estonia** | 1999-2023;  2010-2023 | primary and hospital care | https://statistika.tai.ee/pxweb/en/Andmebaas/Andmebaas__06Ravimistatistika__01Ravimistatistika/ATC-N.px/ | includes all sales of medicines (outpatient and hospitalized sector; OTC drugs; reimbursed and non-reimbursed drugs) |
| **Finland** | 1985-2021;  2017-2020 | primary and hospital care | http://raportit.nam.fi/raportit/kulutus/laakekulutus_e.html | includes all sales of medicines (outpatient and hospitalized sector; OTC drugs; reimbursed and non-reimbursed drugs) |
| **France** | 1995-2022;  not found | primary and hospital care | not found | - |
| **Germany** | 1986-2023;  2012-2023 | primary care | https://arzneimittel.wido.de/PharMaAnalyst/;jsessionid=890879D04043FEF79ED7C0AF1A45B25F?0 | includes reimbursed medication in the outpatient sector for the statuary health insurance (exclusion of OTC drugs, non-reimbursed drugs, hospital sector, private health insurance); consumption of subgroups is provided for the TOP3000 drugs |
| **Greece** | 1998-2004, 2013-2015, 2017-2023; not found | primary care | not found | - |
| **Hungary** | 1995-2023;  not found | primary care | not found | - |
| **Iceland** | 1989-2023;  2015-2024 | primary care | https://app.powerbi.com/view?r=eyJrIjoiZmRiMGJkNmMtZWQ4NC00NmUzLTlkY2UtZTQ0NDk5ZjZmMDE2IiwidCI6Ijc2NGEzMDZkLTBhNjgtNDVhZC05ZjA3LTZmMTgwNDQ0N2NkNCIsImMiOjh9 | includes all sales of medicines of the outpatient sector, also OTC and non-reimbursed drugs (exclusion of hospitalized sector) |
| **Italy** | 2011-2023;  2017-2023 | primary and hospital care | https://www.aifa.gov.it/en/uso-dei-farmaci-in-italia | Includes reimbursed drugs in the outpatient and hospital sector (exclusion of OTC and non-reimbursed drugs) |
| **Latvia** | 2012-2023;  2008-2018 | primary and hospital care | https://www.zva.gov.lv/en/news-and-publications/publications/baltic-statistics-medicines | includes all sales of medicines (outpatient and hospitalized sector; OTC drugs; reimbursed and non-reimbursed drugs) |
| **Lithuania** | 2010-2022;  2010-2022 | primary and hospital care | https://www.zva.gov.lv/en/news-and-publications/publications/baltic-statistics-medicines | includes all sales of medicines (outpatient and hospitalized sector; OTC drugs; reimbursed and non-reimbursed drugs) |
| **Luxembourg** | 2003-2020;  not found | primary care until 2021; primary and hospital care | not found | - |
| **Netherlands** | 2001-2023;  2019-2023 | primary care | https://www.gipdatabank.nl/databank?infotype=g&label=00-totaal&tabel=B_01-basis&geg=ddd&item=N | includes reimbursed medication in the outpatient sector for the statuary health insurance (exclusion of OTC drugs, non-reimbursed drugs, hospital sector, private health insurance) |
| **Norway** | 1999-2023;  2004-2023 | primary care until 2021; primary and hospital care | http://www.norpd.no | includes all sales of medicines (outpatient and hospitalized sector; OTC drugs; reimbursed and non-reimbursed drugs) |
| **Poland** | 2019-2023;  not found | primary care | not found | - |
| **Portugal** | 2000-2023;  not found | primary care | not found | - |
| **Slovakia** | 1996-2022;  not found | primary and hospital care | not found | - |
| **Slovenia** | 2006-2022;  not found | primary care | not found | - |
| **Spain** | 1992-2023;  2010-2023 | primary care until 2018; primary and hospital care | https://www.sanidad.gob.es/areas/farmacia/consumoMedicamentos/ATC/home.htm | includes reimbursed medication in the outpatient sector (exclusion of OTC drugs, non-reimbursed drugs, hospital sector) |
| **Sweden** | 2000-2023;  not found | primary and hospital care | https://www.socialstyrelsen.se/statistik-och-data/statistik/alla-statistikamnen/lakemedel/ | includes all sales of medicines (outpatient and hospitalized sector; OTC drugs; reimbursed and non-reimbursed drugs) |
| **United Kingdom** | 1991-2022;  not found | primary care | not found | - |
| **Croatia** | 2005-2023;  2007-2023 | primary and hospital care | https://www.halmed.hr/en/Promet-proizvodnja-i-inspekcija/Promet/Potrosnja-lijekova/Izvjesca-o-prometu-lijekova/ | includes all sales of medicines (outpatient and hospitalized sector; OTC drugs; reimbursed and non-reimbursed drugs) |

***Table S3:*** *Recent consumption for ATC subgroups of antidepressants. Countries are listed in descending order of the largest consumption in N06AB. Belgium is not listed because the last available data point is from 2014.*

| **country** | **NSMRIs (N06AA)**  **in DID** | **SSRIs**  **(N06AB)**  **in DID** | **MAO-Is (N06AF)**  **in DID** | **MAOA-Is (N06AG)**  **in DID** | **Miscellaneous**  **(N06AX)**  **in DID** |
| --- | --- | --- | --- | --- | --- |
| Iceland | 4.6 | 122.6 | - |  | 41.3 |
| Denmark | 3.5 | 61.6 | 0.10 | 0.00 | 31.0 |
| Spain | 3.4 | 58.8 | 0.00 | 0.01 | 39.4 |
| Finland | 3.9 | 44.6 | - | - | 32.7 |
| Norway | 3.6 | 34.7 | 0.00 | 0.10 | 17.1 |
| Germany | 8.7 | 33.5 | 0.15 | 0.12 | 25.6 |
| Netherlands | 5.8 | 33.5 | 0.50 |  |  |
| Italy | 1.1 | 32.4 | - | - | 13.7 |
| Estonia | 1.9 | 28.2 | 0.01 | 0.00 | 17.4 |
| Lithuania | 1.2 | 25.7 | - | - | 13.2 |
| Croatia | 0.5 | 24.2 | 0.00 | 0.00 | 14.8 |
| Latvia | 1.9 | 10.3 | - |  | 4.0 |

***Table S4:*** *Relative change in consumption for ATC subgroups of AD in the last decade (since 2013). Countries are sorted in descending order of the strongest relative increase of N06AB. Excluded were the countries with insufficient data coverage, including Belgium, Finland, Iceland and Italy.*

| **country** | **NSMRIs (N06AA) relative change** | **SSRIs**  **(N06AB) relative change** | **MAO-Is (N06AF) relative change** | **MAOA-Is (N06AG) relative**  **change** | **Miscellaneous (N06AX) relative**  **change** |
| --- | --- | --- | --- | --- | --- |
| Estonia | -9.5% | 104.3% | 0.0% | 0.0% | 216.4% |
| Lithuania | -14.3% | 61.6% | - | - | 78.4% |
| Latvia | 11.8% | 56.1% | - | - | 100.0% |
| Germany | -20.2% | 43.2% | 25.0% | 20.0% | 48.0% |
| Spain | 30.8% | 28.9% | 0.0% | 0.0% | 97.0% |
| Croatia | -54.5% | 24.1% |  | -100.0% | 142.6% |
| Denmark | -23.9% | 23.0% | 0.0% | 0.0% | 24.0% |
| Norway | 5.9% | -1.7% | 0.0% | 0.0% | 20.4% |

***Table S5:*** *Detailed information on consumption and distributional use for NSMRI (N06AA). Increases are highlighted blue decreases are marked brown and no changes are grey-coloured. “*” indicates non-calculated values due to insufficient data availability. Countries are sorted descending by their last reported consumption.*

| **NSMRIs (N06AA)** | **consumption in DID** | | | | **share in %** | | |
| --- | --- | --- | --- | --- | --- | --- | --- |
| **country** | **DID in 2013** | **last reported DID** | **absolute change since 2013** | **relative change since 2013** | **2013 proportion on consumption** | **last reported proportion** | **change since 2013** |
| Germany | 10.9 | 8.7 | -2.2 | -20.2% | 20.6% | 12.5% | -8.1 % |
| Nether-lands | - | 5.8 | * | * | - | 11.4% | * |
| Iceland | - | 4.6 | * | * | - | 2.7% | * |
| Finland | - | 3.9 | * | * | - | 6.1% | * |
| Belgium | 3.8 | 3.8 | * | * | 5.3% | 5.1% | * |
| Norway | 3.4 | 3.6 | 0.2 | 5.9% | 7.7% | 6.5% | -1.2% |
| Den-mark | 4.6 | 3.5 | -1.1 | -23.9% | 5.8% | 3.6% | -2.2% |
| Spain | 2.6 | 3.4 | 0.8 | 30.8% | 3.9% | 3.4% | -0.5% |
| Estonia | 2.1 | 1.9 | -0.2 | -9.5% | 9.7% | 3.9% | -5.8% |
| Latvia | 1.7 | 1.9 | 0.2 | 11.8% | 16.3% | 11.6% | -4.7% |
| Lithua-nia | 1.4 | 1.2 | -0.2 | -14.3% | 5.6% | 3.0% | -2.6% |
| Italy | - | 1.1 | * | * | - | 2.3% | * |
| Croatia | 1.1 | 0.5 | -0.6 | -54.5% | 4.0% | 1.3% | -2.7% |

***Table S6:*** *Detailed information on consumption and distributional use for SSRI (N06AB). Increases are highlighted blue decreases are marked brown and no changes are grey-coloured. “*” indicates non-calculated values due to insufficient data availability. Countries are sorted descending by their last reported consumption.*

| **SSRIs (N06AB)** | **consumption in DID** | | | | **share in %** | | |
| --- | --- | --- | --- | --- | --- | --- | --- |
| **country** | **DID in 2013** | **last reported**  **DID** | **absolute change since 2013** | **relative change since 2013** | **2013 proportion on consumption** | **last reported proportion** | **change since 2013** |
| Iceland | - | 122.6 | * | * | - | 73.1% | * |
| Denmark | 50.1 | 61.6 | 11.5 | 23.0% | 62.6% | 64.0% | 1.4% |
| Spain | 45.6 | 58.8 | 13.2 | 28.9% | 66.8% | 57.9% | -8.9% |
| Finland | - | 44.6 | * | * | - | 54.7% | * |
| Belgium | 42.3 | 44.1 | * | * | 58.6% | 58.8% | * |
| Norway | 35.3 | 34.7 | -0.6 | -1.7% | 68.4% | 60.8% | -7.6% |
| Germany | 23.4 | 33.5 | 10.1 | 43.2% | 44.1% | 48.5% | 4.4% |
| Netherlands | - | 33.5 | * | * | - | 66.0% | * |
| Italy | - | 32.4 | * | * | - | 68.8% | * |
| Estonia | 13.8 | 28.2 | 14.4 | 104.3% | 64.6% | 59.4% | -5.2% |
| Lithuania | 15.9 | 25.7 | 9.8 | 61.6% | 64.5% | 64.2% | -0.3% |
| Croatia | 19.5 | 24.2 | 4.7 | 24.1% | 73.0% | 61.2% | -11.8% |
| Latvia | 6.6 | 10.3 | 3.7 | 56.1% | 64.2% | 63.9% | -0.3% |

***Table S7:*** *Detailed information on consumption and distributional use for MAO-I (N06AF). Increases are highlighted blue decreases are marked brown and no changes are grey-coloured. “*” indicates non-calculated values due to insufficient data availability. Countries are sorted descending by their last reported consumption. Not all 13 countries provided data, explaining while only eight countries are listed.*

| **MAO-Is (N06AF)** | **consumption in DID** | | | | **share in %** | | |
| --- | --- | --- | --- | --- | --- | --- | --- |
| **country** | **DID in 2013** | **last reported**  **DID** | **absolute change since 2013** | **relative change since 2013** | **2013 proportion on consumption** | **last reported proportion** | **change since 2013** |
| Netherlands | - | 0.50 | * | * | - | 1.0% | * |
| Germany | 0.12 | 0.15 | 0.03 | 25.0% | 0.2% | 0.2% | 0.0% |
| Denmark | 0.10 | 0.10 | 0.00 | 0.0% | 0.1% | 0.1% | 0.0% |
| Estonia | 0.01 | 0.01 | 0.00 | 0.0% | 0.0% | 0.0% | 0.0% |
| Croatia | - | 0.00 | * | * | 0.0% | 0.0% | 0.0% |
| Spain | 0.00 | 0.00 | 0.00 | 0.0% | - | - | * |
| Norway | 0.00 | 0.00 | 0.00 | 0.0% | 0.1% | 0.0% | -0.1% |
| Belgium | 0.00 | 0.00 | * | * | 0.0% | 0.0% | * |

***Table S8:*** *Detailed information on consumption and distributional use for MAOA-I (N06AG). Increases are highlighted blue decreases are marked brown and no changes are grey-coloured. “*” indicates non-calculated values due to insufficient data availability. Countries are sorted descending by their last reported consumption.*

| **MAOA-Is (N06AG)** | **consumption in DID** | | | | **share in %** | | |
| --- | --- | --- | --- | --- | --- | --- | --- |
| **country** | **DID in 2013** | **last reported**  **DID** | **absolute change since 2013** | **relative change since 2013** | **2013 proportion on consumption** | **last reported proportion** | **change since 2013** |
| Belgium | 0.10 | 0.10 | * | * | 0.1% | 0.1% | * |
| Finland | - | 0.34 | * | * | - | 0.4% | * |
| Germany | 0.10 | 0.12 | 0.02 | 20.0% | 0.2% | 0.2% | 0.0% |
| Iceland | - | 0.10 | * | * | - | 0.1% | * |
| Netherlands | - | 0.04 | * | * | - | 0.1% | * |
| Norway | 0.10 | 0.10 | 0.00 | 0.0% | 0.7% | 0.1% | -0.6% |
| Spain | 0.01 | 0.01 | 0.00 | 0.0% | 0.0% | 0.0% | 0.0% |
| Denmark | 0.00 | 0.00 | 0.00 | 0.0% | 0.0% | 0.0% | 0.0% |
| Estonia | 0.00 | 0.00 | 0.00 | 0.0% | 0.0% | 0.0% | 0.0% |
| Croatia | 0.04 | 0.00 | -0.04 | -100.0% | 0.1% | 0.0% | -0.1% |
|  |  |  |  |  |  |  |  |

***Table S9:*** *Detailed information on consumption and distributional use for miscellaneous (N06AX). Increases are highlighted blue decreases are marked brown and no changes are grey-coloured. “*” indicates non-calculated values due to insufficient data availability. Countries are sorted descending by their last reported consumption.*

| **Miscellaneous (N06AX)** | **consumption in DID** | | | | **share in %** | | |
| --- | --- | --- | --- | --- | --- | --- | --- |
| **country** | **DID in 2013** | **last reported**  **DID** | **absolute change since 2013** | **relative change since 2013** | **2013 proportion on consumption** | **last reported proportion** | **change since 2013** |
| Iceland | - | 41.3 | * | * | - | 24.1% | * |
| Spain | 20.0 | 39.4 | 19.4 | 97.0% | 29.4% | 38.8% | 9.4% |
| Finland | - | 32.7 | * | * | - | 40.1% | * |
| Denmark | 25.0 | 31.0 | 6.0 | 24.0% | 31.3% | 32.2% | 0.9% |
| Belgium | 26.0 | 27.0 | * | * | 36.0% | 36.0% | * |
| Germany | 17.3 | 25.6 | 8.3 | 48.0% | 32.6% | 37.0% | 4.4% |
| Estonia | 5.5 | 17.4 | 11.9 | 216.4% | 25.6% | 36.7% | 11.1% |
| Norway | 14.2 | 17.1 | 2.9 | 20.4% | 23.1% | 32.6% | 9.5% |
| Croatia | 6.1 | 14.8 | 8.7 | 142.6% | 22.9% | 37.5% | 14.6% |
| Italy | - | 13.7 | * | * | - | 29.1% | * |
| Lithuania | 7.4 | 13.2 | 5.8 | 78.4% | 29.9% | 32.8% | 2.9% |
| Netherlands | - | 10.9 | * | * | - | 21.5% | * |
| Latvia | 2.0 | 4.0 | 2.0 | 100.0% | 19.5% | 24.5% | 5.0% |

***Table S10:*** *Prediction of DID with ARIMA models for ADs until 2030. Last reported and predicted use, changes and predicted developments are presented. Countries are sorted in descending order of last reported DID.*

| **country** | **last reported DID of ADs** | **predicted DID for ADs in 2030** | **relative change AD consumption** | **absolute change AD consumption** | **predicted development AD consumption** |
| --- | --- | --- | --- | --- | --- |
| Iceland | 164.7 | 195.2 | 18.5% | 30.5 | ↗ |
| Portugal | 154.4 | 190.9 | 23.6% | 36.5 | ↗ |
| United Kingdom | 135.6 | 155.8 | 14.9% | 20.2 | ↗ |
| Sweden | 117.8 | 141.0 | 0.1% | 0.2 | ↗ |
| Spain | 101.5 | 132.7 | 8.7% | 10.7 | ↗ |
| Denmark | 96.2 | 119.9 | 24.6% | 23.7 | ↗ |
| Belgium | 88.2 | 105.9 | 20.1% | 17.7 | ↗ |
| Finland | 85.2 | 123.5 | 45.0% | 38.3 | ↗ |
| Greece | 76.5 | 84.8 | 10.9% | 8.3 | ↗ |
| Czech Republic | 73.3 | 104.7 | 42.9% | 31.4 | ↗ |
| Germany | 69.1 | 81.3 | 17.7% | 12.2 | ↗ |
| Slovenia | 68.4 | 87.6 | 28.1% | 19.2 | ↗ |
| Norway | 63.5 | 61.7 | -2.8% | -1.8 | ↘ |
| Austria | 63.1 | 49.4 | -21.7% | -13.7 | ↘ |
| France | 61.1 | 71.0 | 16.2% | 9.9 | ↗ |
| Luxembourg | 53.1 | 51.3 | -3.4% | -1.8 | ↘ |
| Netherlands | 50.8 | 57.1 | 12.5% | 6.3 | ↗ |
| Estonia | 47.5 | 73.9 | 55.5% | 26.4 | ↗ |
| Italy | 47.1 | 54.6 | 15.9% | 7.5 | ↗ |
| Slovakia | 47.1 | 47.3 | 0.4% | 0.2 | ↗ |
| Lithuania | 40.1 | 54.3 | 35.4% | 14.2 | ↗ |
| Croatia | 39.4 | 49.2 | 24.8% | 9.8 | ↗ |
| Poland | 39.1 | - | - | - | - |
| Hungary | 30.5 | 26.0 | -14.6% | -4.5 | ↘ |
| Latvia | 26.3 | 47.6 | 80.9% | 21.3 | ↗ |
| Median of the country values | 63.5 | 77.6 | 16.6% | 10.3 | ↗ |

***Table S11:*** *Forecast of DID prescriptions for ADs (ATC N06A) until 2030 with ARIMA models. Countries are sorted alphabetically.*

| **country** | **2022** | **2023** | **2024** | **2025** | **2026** | **2027** | **2028** | **2029** | **2030** |
| --- | --- | --- | --- | --- | --- | --- | --- | --- | --- |
| **Austria** |  | 62.41 | 61.43 | 60.15 | 58.59 | 56.74 | 54.59 | 52.15 | 49.43 |
| **Belgium** |  | 90.42 | 92.63 | 94.85 | 97.07 | 99.29 | 101.5 | 103.72 | 105.94 |
| **Croatia** |  |  | 40.84 | 42.23 | 43.63 | 45.02 | 46.42 | 47.82 | 49.21 |
| **Czech Republic** |  | 76.6 | 80.47 | 84.14 | 88.12 | 92.07 | 96.21 | 100.4 | 104.73 |
| **Denmark** |  |  |  | 100.18 | 104.15 | 108.1 | 112.04 | 115.96 | 119.86 |
| **Estonia** |  |  | 50.94 | 54.49 | 58.15 | 61.92 | 65.79 | 69.78 | 73.88 |
| **Finland** | 89.01 | 92.94 | 96.97 | 101.12 | 105.38 | 109.75 | 114.23 | 118.82 | 123.53 |
| **France** |  | 62.33 | 63.57 | 64.8 | 66.04 | 67.27 | 68.51 | 69.74 | 70.98 |
| **Germany** |  |  | 71.23 | 73.02 | 74.71 | 76.36 | 78.01 | 79.66 | 81.31 |
| **Greece** |  |  | 79.95 | 81.58 | 82.78 | 83.59 | 83.55 | 84.37 | 84.81 |
| **Hungary** |  |  | 30.07 | 29.59 | 29.01 | 28.33 | 27.75 | 26.93 | 26.04 |
| **Iceland** |  |  | 169.06 | 173.42 | 177.78 | 182.14 | 186.5 | 190.86 | 195.22 |
| **Italy** |  |  | 48.4 | 49.62 | 50.67 | 51.69 | 52.67 | 53.64 | 54.58 |
| **Latvia** |  |  | 29.3 | 32.03 | 34.73 | 38.15 | 40.79 | 44.44 | 47.57 |
| **Lithuania** |  | 41.45 | 44.68 | 45.66 | 47.7 | 49.19 | 50.96 | 52.58 | 54.28 |
| **Luxembourg** |  | 52.74 | 52.49 | 52.25 | 52.18 | 51.96 | 51.74 | 51.53 | 51.32 |
| **Netherlands** |  |  | 51.7 | 52.61 | 53.51 | 54.42 | 55.32 | 56.23 | 57.13 |
| **Norway** |  |  | 63.74 | 63.81 | 63.72 | 63.46 | 63.05 | 62.46 | 61.72 |
| **Poland** | model cannot be built | | | | | | | | |
| **Portugal** |  |  | 159.61 | 164.83 | 170.04 | 175.25 | 180.47 | 185.68 | 190.9 |
| **Slovakia** |  | 48.3 | 48.59 | 48.36 | 48.14 | 47.92 | 47.71 | 47.49 | 47.28 |
| **Slovenia** |  | 70.80 | 73.20 | 75.60 | 78.00 | 80.40 | 82.80 | 85.20 | 87.60 |
| **Spain** |  |  | 104.43 | 107.37 | 110.3 | 113.23 | 116.17 | 119.1 | 122.03 |
| **Sweden** |  |  | 121.22 | 124.6 | 127.93 | 131.22 | 134.47 | 137.67 | 140.83 |
| **United**  **Kingdom** |  |  | 138.09 | 140.59 | 145.62 | 148.15 | 150.68 | 135.22 | 155.78 |

***Table S12:*** *Fit metrics for ARIMA models and defined outliers. Time breaks reported by the OECD [20, 21] were defined as a level shift. An innovational outlier was defined for each country in the beginning of the COVID pandemic in 2020. Green coloured are fit metrics that are considered good, yellow-coloured being moderate and orange-coloured being poor (see section “Materials and Methods”). Countries are sorted alphabetically.*

| **country** | **years with outlier** | **ARIMA(q,d,q) model** | **Stationary**  **R-squared** | **R-squared** | **RMSE** | **MAPE** | **MAE** | **MaxAPE** | **MaxAE** | **Normalized BIC** |
| --- | --- | --- | --- | --- | --- | --- | --- | --- | --- | --- |
| **Austria** | 2020 | (0, 2, 0) | 1.776E-15 | 0.809 | 0.696 | 0.938 | 0.572 | 2.417 | 1.409 | -0.506 |
| **Belgium** | 2008, 2020 | (0, 1, 0) | 0.352 | 0.996 | 1.079 | 1.392 | 0.775 | 3.593 | 2.417 | 0.538 |
| **Croatia** | 2013, 2020 | (0, 1, 0) | 0.002 | 0.968 | 1.376 | 3.880 | 0.814 | 24.180 | 3.796 | 1.120 |
| **Czech Republic** | 2020 | (1, 2, 0) | 0.477 | 0.998 | 1.157 | 6.774 | 0.818 | 29.510 | 3.356 | 0.563 |
| **Denmark** | 2020 | (0, 2, 0) | 0.006 | 0.988 | 1.915 | 2.177 | 1.371 | 7.657 | 4.484 | 1.550 |
| **Estonia** | 2020 | (0, 2, 1) | 0.297 | 0.997 | 0.713 | 2.907 | 0.556 | 9.262 | 1.149 | -0.269 |
| **Finland** | 2006, 2020 | (0, 2, 0) | 0.032 | 0.998 | 1.147 | 2.454 | 0.761 | 8.285 | 3.288 | 0.578 |
| **France** | 2020 | (0, 1, 0) | 0.020 | 0.958 | 1.577 | 2.752 | 1.330 | 5.676 | 2.935 | 1.156 |
| **Germany** | 2010, 2020 | (1, 1, 0) | 0.218 | 0.997 | 1.222 | 4.776 | 0.915 | 23.398 | 2.673 | 0.792 |
| **Greece** | 2004, 2018, 2020 | (3, 0, 1) | 0.859 | 0.859 | 10.510 | 18.623 | 3.629 | 215.058 | 24.947 | 6.038 |
| **Hungary** | 2013, 2020 | (2, 0, 1) | 0.98 | 0.980 | 1.161 | 5.390 | 0.711 | 54.420 | 3.428 | 0.995 |
| **Iceland** | 2007, 2020 | (0, 1, 0) | 0.032 | 0.995 | 3.470 | 5.015 | 2.738 | 26.864 | 8.059 | 2.800 |
| **Italy** | 2011, 2020 | (1, 1, 0) | 0.521 | 0.989 | 0.348 | 0.525 | 0.215 | 1.380 | 0.563 | -1.282 |
| **Latvia** | 2020 | (2, 2, 2) | 0.931 | 0.999 | 0.230 | 0.745 | 0.113 | 2.129 | 0.262 | -1.555 |
| **Lithuania** | 2020 | (2, 1, 2) | 0.824 | 0.993 | 0.712 | 1.426 | 0.406 | 3.708 | 0.889 | 0.563 |
| **Luxem-bourg** | 2020 | (1, 0, 0) | 0.719 | 0.719 | 3.169 | 4.472 | 2.008 | 26.329 | 9.531 | 2.772 |
| **Nether-lands** | 2020 | (0, 1, 0) | 0.010 | 0.949 | 1.102 | 1.742 | 0.687 | 7.560 | 2.805 | 0.475 |
| **Norway** | 2020 | (0, 2, 0) | 0.012 | 0.889 | 1.436 | 1.778 | 0.994 | 7.527 | 3.936 | 0.997 |
| **Poland** | 2020 | (1, 2, 0) | model cannot be built | | | | | | | |
| **Portugal** | 2020 | (0, 1, 0) | 0.020 | 0.992 | 3.151 | 2.726 | 2.162 | 8.738 | 9.186 | 2.568 |
| **Slovakia** | 2020 | (1, 0, 2) | 0.888 | 0.888 | 5.102 | 25.996 | 2.057 | 532.489 | 22.365 | 3.870 |
| **Slovenia** | 2016, 2020 | (0, 1, 0) | 0.224 | 0.988 | 1.071 | 1.441 | 0.688 | 5.898 | 2.200 | 0.658 |
| **Spain** | 2020 | (0, 1, 0) | 0.002 | 0.997 | 1.479 | 2.791 | 1.084 | 16.224 | 3.167 | 1.004 |
| **Sweden** | 2014, 2020 | (0, 2, 1) | 0.339 | 0.988 | 2.137 | 2.042 | 1.515 | 6.735 | 4.189 | 2.081 |
| **United Kingdom** | 2020 | (1, 1, 0) | 0.181 | 0.996 | 2.575 | 3.091 | 1.636 | 13.888 | 7.402 | 2.224 |

***Table S13:*** *Assessment of ARIMA forecast in regard of model reliability, graded in good (green-coloured), moderate (yellow-coloured) and poor (orange-coloured). Fit metrics are considered good for at least three good values, moderate with at least three good or moderate values; and poor for two and more poor values. Regarding the relative range of the 95% confidence interval, under 20% is good, between 20-50% is moderate and exceeding 50% is poor. In parentheses, the DID prescriptions for the lower and upper confidence interval are provided. Countries are sorted alphabetically.*

| **country** | **fit metrics** | **relative range UCL and LCL prediction in 2030 [UCL;LCL]** |
| --- | --- | --- |
| **Austria** | moderate | 89.7% [71.59; 27.27] |
| **Belgium** | moderate | 12.0% [112.27; 99.61] |
| **Croatia** | moderate | 31.5% [56.97; 41.46] |
| **Czech Republic** | moderate | 41.2% [126.3; 83.15] |
| **Denmark** | moderate | 62.9% [157.56; 82.17] |
| **Estonia** | moderate | 26.4% [83.62; 64.13] |
| **Finland** | moderate | 63.8% [162.96; 84.1] |
| **France** | moderate | 25.9% [80.17; 61.79] |
| **Germany** | moderate | 21.3% [89.97; 72.64] |
| **Greece** | moderate | 67.2% [113.32; 56.29] |
| **Hungary** | moderate | 37.0% [30.86; 21.22] |
| **Iceland** | moderate | 19.2% [213.94; 176.49] |
| **Italy** | good | 22.1% [60.56; 48.6] |
| **Latvia** | good | 18.8% [52.05; 43.09] |
| **Lithuania** | good | 7.8% [56.41; 52.15] |
| **Luxembourg** | good | 44.9% [62.85; 39.79] |
| **Netherlands** | moderate | 21.3% [63.22; 51.05] |
| **Norway** | moderate | 114.5% [97.06; 26.37] |
| **Poland** | model cannot be built | - |
| **Portugal** | moderate | 18.2% [208.23; 173.56] |
| **Slovakia** | moderate | 75.7% [65.16; 29.39] |
| **Slovenia** | moderate | 15.0% [94.15; 81.05] |
| **Spain** | moderate | 13.1% [130.04; 114.03] |
| **Sweden** | moderate | 30.0% [161.97; 119.7] |
| **United Kingdom** | moderate | 27.8% [184.47; 139.4] |

Supplemental Figures

***Fig. S1:*** *Applied regional classification of the analysed European countries. Countries not included in the analysis are shown in grey. The classification is partly based on geographical considerations and was structured to ensure that each region includes more than three countries and that regions contain a broadly comparable number of countries. Alternative regional classifications may be possible.*


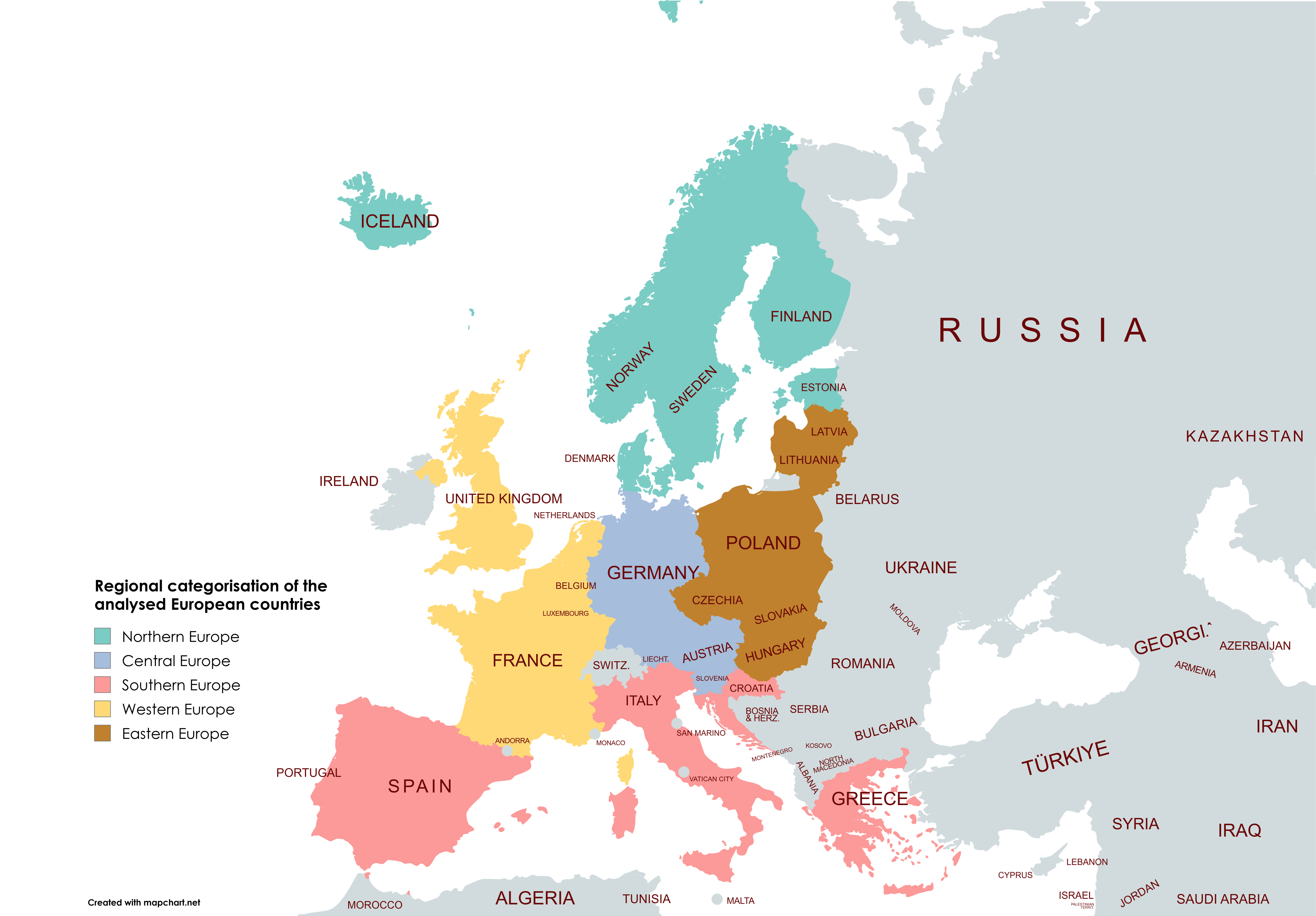


***Fig. S2:*** *Proportional use of NSMRIs (N06AA), SSRIs (N06AB), MAO-Is (N06AF), MAOA-Is (N06AG) and miscellaneous (N06AX) for countries with publicly available data.*


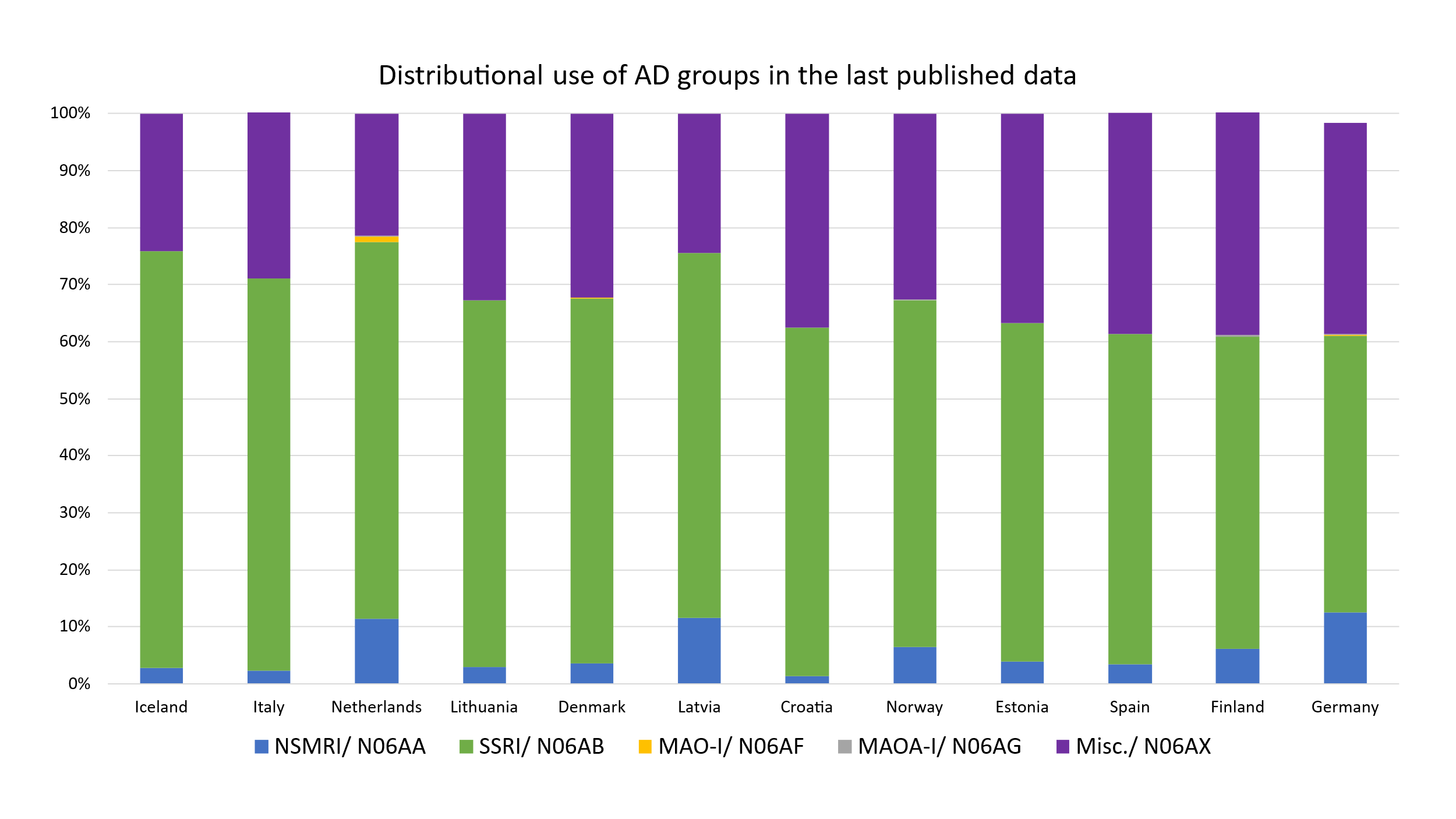


***Fig. S3:*** *Geographical distribution of ATC subgroup consumption in the analysed countries. The most recently reported prescriptions in DID are projected. Consumption ranges are highlighted in colour: dark brown for below average, light brown for slightly below average, yellow for average, light blue for slightly above average and dark blue for above average. Countries for which no data were available are shown in grey. Depicted are the subgroups NSMRIs (a), SSRIs (b), MAO-Is (c), MAOA-Is (d), and miscellaneous. (e).*


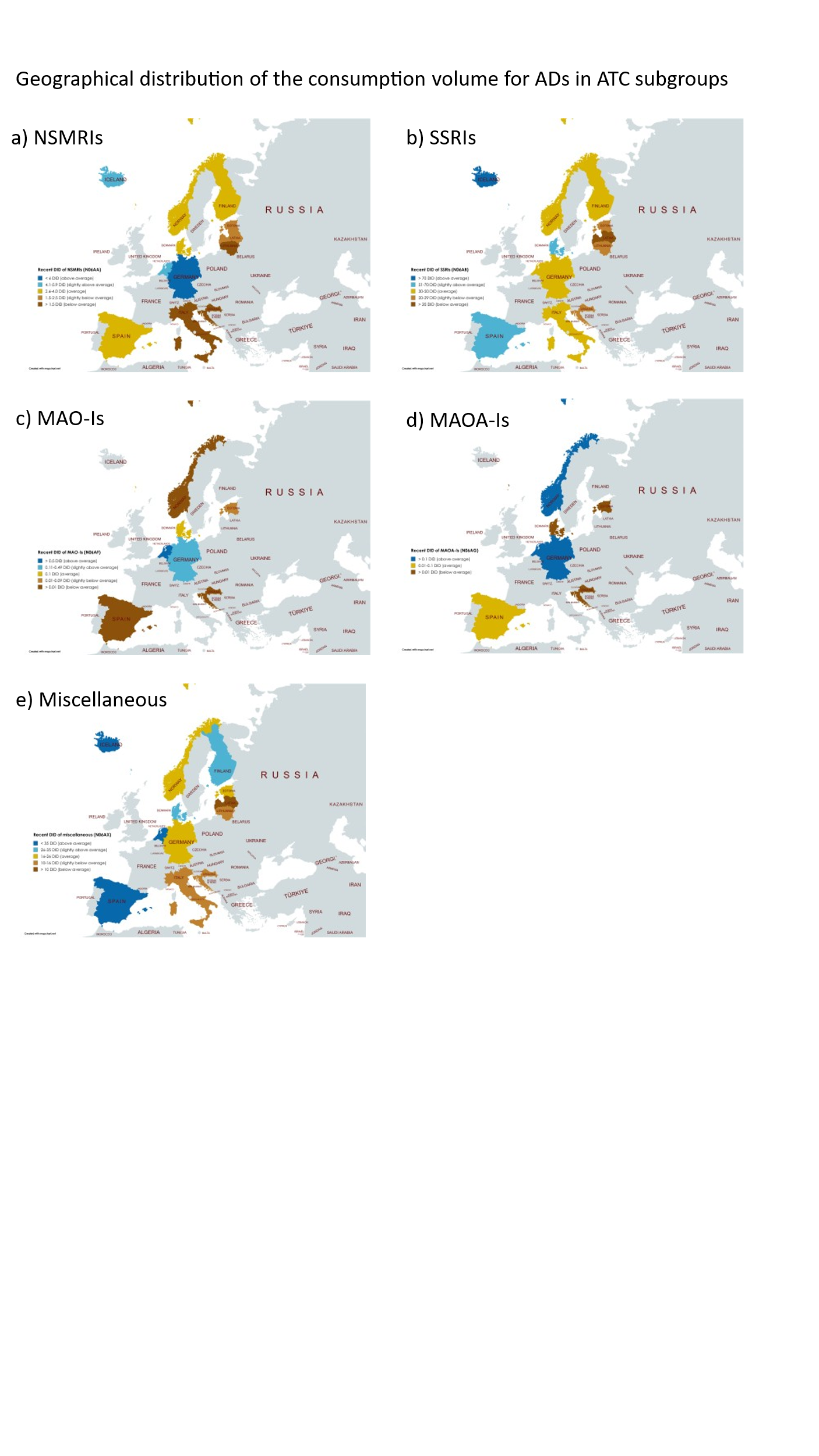

Supplement: Supplementary file 1 — Supplementary file1 (DOCX 2736 kb) [file 11096_2025_2078_MOESM1_ESM.docx]
